# Predictors of the Home-Clinic Blood Pressure Difference: A Systematic Review and Meta-Analysis

**DOI:** 10.1093/ajh/hpv157

**Published:** 2015-09-22

**Authors:** James P. Sheppard, Ben Fletcher, Paramjit Gill, Una Martin, Nia Roberts, Richard J. McManus

**Affiliations:** ^1^Nuffield Department of Primary Care Health Sciences, University of Oxford, Oxford, UK;; ^2^Primary Care Clinical Sciences, University of Birmingham, Birmingham, UK;; ^3^School of Clinical and Experimental Medicine, University of Birmingham, Birmingham, UK;; ^4^Bodleian Healthcare Libraries, Knowledge Centre, University of Oxford, Oxford, UK.

**Keywords:** ambulatory blood pressure monitoring, hypertension, masked hypertension, primary care, white coat hypertension.

## Abstract

**BACKGROUND:**

Patients may have lower (white coat hypertension) or higher (masked hypertension) blood pressure (BP) at home compared to the clinic, resulting in misdiagnosis and suboptimal management of hypertension. This study aimed to systematically review the literature and establish the most important predictors of the home-clinic BP difference.

**METHODS:**

A systematic review was conducted using a MEDLINE search strategy, adapted for use in 6 literature databases. Studies examining factors that predict the home-clinic BP difference were included in the review. Odds ratios (ORs) describing the association between patient characteristics and white coat or masked hypertension were extracted and entered into a random-effects meta-analysis.

**RESULTS:**

The search strategy identified 3,743 articles of which 70 were eligible for this review. Studies examined a total of 86,167 patients (47% female) and reported a total of 60 significant predictors of the home-clinic BP difference. Masked hypertension was associated with male sex (OR 1.47, 95% confidence interval (CI) 1.18–1.75), body mass index (BMI, per kg/m^2^ increase, OR 1.07, 95% CI 1.01–1.14), current smoking status (OR 1.32, 95% CI 1.13–1.50), and systolic clinic BP (per mm Hg increase, OR 1.10, 95% CI 1.01–1.19). Female sex was the only significant predictor of white coat hypertension (OR 3.38, 95% CI 1.64–6.96).

**CONCLUSIONS:**

There are a number of common patient characteristics that predict the home-clinic BP difference, in particular for people with masked hypertension. There is scope to incorporate such predictors into a clinical prediction tool which could be used to identify those patients displaying a significant masked or white coat effect in routine clinical practice.

Hypertension is an important risk factor for cardiovascular disease,^1^ the major cause of morbidity and mortality worldwide.^2^ Effective diagnosis and management of hypertension depends on accurate measurement of blood pressure, which allows appropriate targeting of antihypertensive treatment. Ambulatory blood pressure monitoring (ABPM) is considered to be the “gold standard” measure of blood pressure, because multiple readings are taken and because it is associated with a range of cardiovascular outcomes and end organ damage.^3–7^ Ambulatory blood pressure is usually lower than clinic blood pressure^8–11^ due to the white coat effect (Table 1),^12^ and as such, clinical guidelines recommend that ABPM (or home) blood pressure targets are 5mm Hg lower than the corresponding clinic values.^13,14^ However, this “home-clinic blood pressure difference” is not always consistent. In some patients, blood pressures measured at home or with ABPM are higher than would be expected for the corresponding clinic blood pressure, the so-called masked effect (Table 1).^15^ Such patients are likely to be undertreated and have increased target organ damage^16,17^ with subsequent increased cardiovascular mortality compared to normotensive patients.^18,19^

**Table 1.** Definitions of the home-clinic blood pressure difference

Term	Definition
Home-clinic blood pressure difference	The difference between blood pressure measured with ABPM or at home (self-monitored) and blood pressure measured in the clinic.
White coat effect	A negative home-clinic blood pressure difference. Blood pressure measured with ABPM (or at home) is *lower* than the corresponding clinic blood pressure.
White coat hypertension	A negative home-clinic blood pressure difference. Blood pressure measured with ABPM (or at home) is <135/85mm Hg but the corresponding clinic blood pressure is ≥140/90mm Hg.
Masked effect	A positive home-clinic blood pressure difference. Blood pressure measured with ABPM (or at home) is *higher* than the corresponding clinic blood pressure.
Masked hypertension	A positive home-clinic blood pressure difference. Blood pressure measured with ABPM (or at home) is ≥135/85mm Hg but the corresponding clinic blood pressure is <140/90mm Hg.
Masked uncontrolled hypertension	A positive home-clinic blood pressure difference in patients with a previous diagnosis of hypertension. Blood pressure measured with ABPM (or at home) is ≥135/85mm Hg but the corresponding clinic blood pressure is <140/90mm Hg (incorrectly suggesting the patient is controlled).

Abbreviation: ABPM, ambulatory blood pressure monitoring.

Clinic blood pressure monitoring is still recommended for initial screening of blood pressure in routine clinical practice,^13,14^ and thus, identifying those patients most likely to display a white coat or masked effect is important to avoid misdiagnosis and mismanagement of hypertension. There is a large body of literature proposing factors that predict white coat or masked hypertension,^20–22^ but no studies have systematically reviewed the evidence. Consequently there is little consensus as to which factors are most important or how they should be used in clinical practice to guide diagnosis and management decisions. The present study aimed to systematically review the literature and establish the most important predictors of a significant home-clinic blood pressure difference to inform interventions that might identify those with discordant clinic and ambulatory blood pressure in routine clinical practice.

## METHODS

This study systematically reviewed all existing literature examining factors that predict the home-clinic blood pressure difference. The protocol is available in the Supplementary Appendix.

### Search strategy

A scoping search was carried out to identify background literature and provide an estimate of the volume of literature on the topic. A search strategy (see Supplementary Appendix) was then designed for use with MEDLINE and then adapted to run across the following databases: CINAHL (EBSCO), The Cochrane (Wiley) CENTRAL Register of Controlled Trials, EMBASE (Ovid), MEDLINE (Ovid) and MEDLINE In Process (Ovid), Science Citation Index – Expanded & Conference Proceedings Citation Index – Science, and The ZETOC (Mimas) database. Searches were carried out up to and including March 2014. In order to capture as broad a range of studies as possible, no language or date limits were applied, although animal studies, letters, comments, and review articles were excluded. In addition to searches of electronic databases, reference lists of studies included in the review were checked to identify any further relevant papers.

### Selection of studies and inclusion criteria

Two authors (J.P.S. and B.F.) reviewed the titles (10% independently) and abstracts (100% independently) of potentially relevant articles for inclusion. Studies were selected for full document screening and data extraction based on the following criteria:

- Included a measure out-of-office blood pressure (home or ambulatory blood pressure).
- Included a measure of clinic blood pressure.
- A cross-sectional study examining data from a single time point.
- Examined independent variables routinely available or measurable in a primary care clinic setting.
- Examined the association between these variables and the home-clinic blood pressure difference, white coat or masked hypertension (outcome variable).
- Included primary data.

The review aimed to identify factors that could be utilized by clinicians in the routine diagnosis and management of hypertension in a Primary Care setting. Thus, studies were excluded from the review if they:

- Examined patients in hospital for surgery or treatment for a specialist condition (e.g., haemodialysis, pregnancy)
- Examined measurements taken in a nonclinical or pharmacy setting.
- Studied patients aged below 18 years.

### Data collection

Data were extracted from all relevant articles identified in the search strategy by J.P.S. and B.F. This included the study setting and population, basic patient demographics, clinic blood pressure, out-of-office blood pressure, and the outcome of interest (home-clinic blood pressure difference, white coat or masked effect, white coat or masked hypertension). Where a logistic regression analysis was performed examining the association between specific variables and the home-clinic blood pressure difference, relevant odds ratios (ORs) for each predictor of this difference were extracted. The form used for data extraction is available in the Supplementary Appendix.

During data extraction, the methodological quality and risk of bias of individual studies were assessed. This quality assessment covered domains of selection bias, detection bias, accuracy of measurement, analysis, and adjustment for confounding using a combination of questions from the QUADAS-2^23^ and CASP^24^ checklists for the assessment of cohort studies.

### Statistical analysis

The primary outcome of this review was to identify the most important factors that predict a significant home-clinic blood pressure difference. This was defined by (a) the number of studies citing specific risk factors for the home-clinic blood pressure difference, white coat or masked hypertension and (b) a pooled OR for the most commonly cited predictors of white coat or masked hypertension. This pooled estimate was based on log OR estimates and their confidence intervals (CIs) synthesized in a random-effects meta-analysis using the method of DerSimonian and Laird.^25^ This method allows for between-study heterogeneity in the true ORs and produces a pooled estimate and 95% CIs to summarize the association between independent predictors and white coat or masked hypertension. Where 95% CIs were not presented in an included article, they were estimated from the corresponding *P* values using the methods described by Altman and Bland.^26^

Sensitivity analyses were conducted focusing on those high quality studies that identified and corrected their analysis for confounding variables including age and sex. Where sufficient data were available, further sensitivity analyses explored the association between independent predictors and white coat or masked hypertension defined according to ambulatory blood pressure (daytime or 24 hour) or home monitoring and in subgroup populations: unselected patients and those with diagnosed hypertension (in patients with hypertension, studies examined predictors of white coat hypertension or masked uncontrolled hypertension).^27^

All analyses were conducted using STATA version 13.1 (MP parallel edition, StataCorp, College Station, TX). Data are presented as proportions of the total study population, means with SD or ORs with 95% CIs unless otherwise stated.

## RESULTS

**Figure 1.**
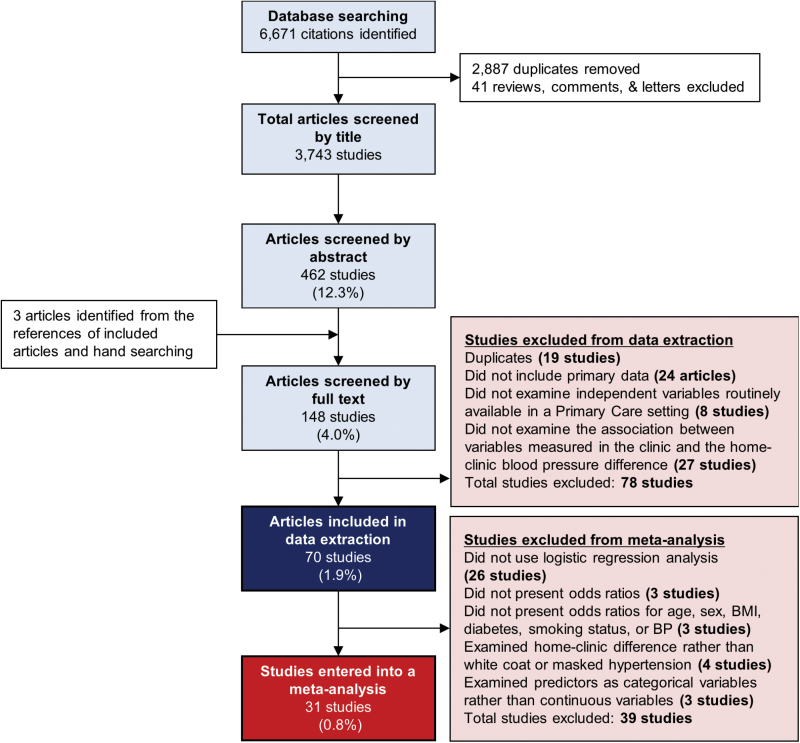
Screening and selection of studies to include in analysis of predictors of the home-clinic blood pressure difference. Abbreviations: BMI, body mass index; sBP, systolic blood pressure; dBP, diastolic blood pressure.

The search strategy identified 3,743 unique articles of which 70 were eligible for this review after title, abstract, and full text screening (Figure 1). Studies were conducted in 27 different countries in a community, primary care or hospital outpatient setting (Table 2). A total of 86,167 patients (mean age 54.5 years) were examined, including 40,622 females (47%) and 40,840 patients on antihypertensive treatment. Study populations varied from unselected cohorts to those with normotension, hypertension, diabetes, or chronic kidney disease.

**Table 2.** Characteristics of included studies

Author	Year	Country	Setting	Population	Sample size	Mean age (years)	Sex (% female)	Out-of-office monitoring	Outcome of interest
Abir-Khalil *et al*.	2009	Morocco	Outpatient clinic	Admitted to cardiology unit	2,462	50.5	58%	ABPM	White coat hypertension
Afsar *et al*.	2013	Turkey	Outpatient clinic	Diabetic	102	48.9	61%	ABPM	Masked hypertension
Akilli *et al*.	2014	Turkey	Outpatient clinic	Diabetic	85	50.7	41%	ABPM	Masked hypertension
Andalib *et al*.	2010	Canada	Primary Care	Hypertensives	2,728	60.3	55%	Home	Masked hypertension
Asayama *et al*.	2009	Japan	Community	Unselected	395	63.5	70%	Home	Masked hypertension
Azizi *et al*.	2013	Morocco	Outpatient clinic	Normotensives	438	47.3	49%	ABPM	Masked hypertension
Bakalakou *et al*.	2013	Greece	n/a	Hypertensives	305	57.2	59%	ABPM	Masked nocturnal hypertension
Barochiner *et al*.	2013	Argentina	Outpatient clinic	Hypertensives	172	64.8	69%	Home	Masked hypertension
Ben-Dov *et al*.	2007a	Israel	Outpatient clinic	Referred for ABPM	3,928	55.1	53%	ABPM	Home-clinic difference
Ben-Dov *et al*.	2007b	Israel	Outpatient clinic	Referred for ABPM	3,957	54.8	58%	ABPM	White coat and masked hypertension
Bucio *et al*.	2011	Mexico	Outpatient clinic	Unselected	49	40.9	53%	ABPM	White coat hypertension
Cacciolati *et al*.	2011	France	Community	Unselected	690	78.8	65%	Home	Masked hypertension
Calvo-Vargas *et al*.	1999	Mexico	Outpatient clinic	n/a	243	56.5	80 %	Home	Home-clinic difference
Charvat *et al*.	2010	Czech Rep.	n/a	Diabetic	64	—	—	ABPM	Masked hypertension
Dolan *et al*.	2004	Ireland	Outpatient clinic	Referred for ABPM	5,716	53.6	53%	ABPM	White coat hypertension
Florian *et al*.	2013	USA	Community	Unselected	1,652	—	—	Home	Masked hypertension
Gorostidi *et al*.	2013	Spain	Primary Care/ clinic	Chronic kidney disease	5,693	67.0	42%	ABPM	White coat and masked hypertension
Gualdiero *et al*.	2000	UK	Outpatient clinic	Referred for ABPM	1,553	53.4	49%	ABPM	Home-clinic difference
Hanninen *et al*.	2011	Finland	Community	Unselected	1,459	55.8	53%	Home	Masked hypertension
Hermida *et al*.	2004	Spain	n/a	Hypertensives	837	49.5	51%	ABPM	Home-clinic difference
Hernández del Ray	1996	Spain	Outpatient clinic	Hypertensives	106	43.0	52%	ABPM	White coat hypertension
Hiraizumi *et al*.	1998	Japan	n/a	Patients with raised office BP	86	—	62%	ABPM	Home-clinic difference
Horikawa *et al*.	2008	Japan	Primary Care	Hypertensives	3,308	66.2	56%	Home	Home-clinic difference
Hozawa *et al*.	2001	Japan	Community	Unselected	1,789	—	—	Home	Home-clinic difference
Huang *et al*.	2010	Taiwan	Outpatient clinic	Hypertensives	121	45.7	37%	ABPM	Home-clinic difference
Hwang *et al*.	2007	Korea	Outpatient clinic	Referred for ABPM	967	51.9	48%	ABPM	White coat and masked hypertension
Iimuro *et al*.	2013	Japan	Outpatient clinic	Chronic kidney disease	1,075	60.7	37%	ABPM	Home-clinic difference
Ishikawa *et al*.	2007	Japan	Outpatient clinic	Hypertensives	405	66.9	45%	Home	Masked (morning) hypertension
Jhalani *et al*.	2005	USA	Outpatient clinic	Hypertensives	226	52.0	53%	ABPM	Home-clinic difference
Kabutoya *et al*.	2009	Japan	Outpatient clinic	Hypertensives	969	66.5	58%	Home	Home-clinic difference
Kayrak *et al*.	2010	Turkey	Outpatient clinic	Ungoing exercise testing	61	47.3	21%	ABPM	Masked hypertension
Kim *et al*.	2011	Korea	Community	Normotensives	84	33.1	37%	ABPM	Masked hypertension
Koupil *et al*.	2005	Sweden	Community	Unselected (aged ~70 years)	736	70.9	0%	ABPM	White coat and masked hypertension
Labinson *et al*.	2008	USA	Primary Care	Patients with raised office BP	65	54.0	55%	ABPM	Home-clinic difference
Lee *et al*.	2008	Korea	Primary Care	Hypertensives	4,435	57.1	51%	Home	Masked hypertension
Lerman *et al*.	1989	USA	Primary Care	Hypertensives	98	54.6	43%	ABPM	Home-clinic difference
Lindbaek *et al*.	2003	Norway	Primary Care	Suspected/treated hypertension	221	58.0	48%	ABPM	Home-clinic difference
MacDonald *et al*.	1999	Canada	Outpatient clinic	Hypertensives	103	59.3	47%	ABPM	White coat hypertension
Mallion *et al*.	2006	France	Primary Care	Hypertensives	1,150	69.0	63%	Home	Masked hypertension
Manios *et al*.	2008	Greece	Outpatient clinic	Unselected	2,004	50.9	53%	ABPM	Home-clinic difference
Mansoor *et al*.	1996	USA	Outpatient clinic	Hypertensives	64	56.0	64%	ABPM	Home-clinic difference
Markis *et al*.	2009	Greece	Outpatient clinic	Unselected	254	55.0	60%	ABPM	Masked hypertension
Martinez *et al*.	1999	Spain	Primary Care	Hypertensives	345	51.8	52%	ABPM	White coat hypertension
Nasothimiou *et al*.	2012	Greece	Outpatient clinic	Referred for ABPM	613	53.0	43%	ABPM/Home	White coat and masked hypertension
Niiranen *et al*.	2006	Finland	Community	Unselected	1,440	55.0	53%	Home	White coat hypertension
Obara *et al*.	2005	Japan	Primary Care	Hypertensives	3,400	66.2	55%	Home	White coat and masked hypertension
Parati *et al*.	2012	Worldwide	Outpatient clinic	Unselected	9,753	56.0	51%	ABPM	Masked hypertension
Park *et al*.	2011	Korea	Outpatient clinic	Hypertensives	511	57.2	55%	Home	Masked hypertension
Rassmussen *et al*.	1998	Denmark	Outpatient clinic	Unselected	1,855	—	48%	ABPM	Home-clinic difference
Rodrigues *et al*.	2009	Brazil	n/a	Diabetic	566	49.1	47%	ABPM	Home-clinic difference
Sandvik *et al*.	1998	Norway	Primary Care	Hypertensives	75	50.1	65%	Home	White coat hypertension
Schoenthaler *et al*.	2010	USA	Community	Normotensives	240	35.9	61%	ABPM	(Marked) masked hypertension
Sheppard *et al*.	2014	UK	Primary Care	Hypertensives	220	67.0	53%	Home	White coat/masked effect
Smirnova *et al*.	2009	Russia	n/a	Hypertensives	39	53.7	51%	ABPM	Home-clinic difference
Sobrino *et al*.	2013	Spain	Outpatient clinic	Normotensives	485	43.1	55%	ABPM	Masked hypertension
Sobrino *et al*.	2011	Spain	Outpatient clinic	Hypertensives	302	56.2	56%	ABPM	Masked hypertension
Spruill *et al*.	2007	USA	Outpatient clinic	Unselected	214	51.7	55%	ABPM	Home-clinic difference
Streitel *et al*.	2011	USA	Outpatient clinic	Unselected	252	45.2	53%	ABPM	Home-clinic difference
Sung *et al*.	2013	Taiwan	Community	Unselected	1,257	53.0	47%	ABPM	Home-clinic difference
Tam *et al*.	2007	Hong Kong	Primary Care	Referred for ABPM	617	52.9	—	ABPM	White coat hypertension
Tardif *et al*.	2009	Canada	Primary Care	Hypertensives	3,247	—	—	Home	Masked hypertension
Thomas *et al*.	2012	UK	Outpatient clinic	Unselected	2,381	56.0	53%	ABPM	Home-clinic difference
Trudel *et al*.	2009	Canada	Community	Unselected	2,370	44.0	61%	ABPM	White coat and masked hypertension
Tsai *et al*.	2003	Taiwan	n/a	Unselected	41	42.6	59%	ABPM	Home-clinic difference
Uze *et al*.	2012	Japan	Outpatient clinic	Diabetic	193	62.7	55%	ABPM	Masked hypertension
Verdecchia *et al*.	2001	Italy	Outpatient clinic	Hypertensives	1,546	39.0	34%	ABPM	White coat hypertension
Wang *et al*.	2007	China	Community	Unselected	694	48.5	54%	ABPM	White coat and masked hypertension
Wing *et al*.	2002	Australia	Primary Care	Hypertensives	713	72.0	47%	ABPM	Masked hypertension
Yoon *et al*.	2012	Korea	Outpatient clinic	Hypertensives	1,087	57.0	52%	Home	Home-clinic difference
Zhou *et al*.	2013	China	Outpatient clinic	Diabetic	856	45.1	45%	ABPM	Masked hypertension

References mentioned in the table are found in the Supplementary Appendix.

Abbreviations: ABPM, ambulatory blood pressure monitoring; Home, home blood pressure monitoring; BP, blood pressure.

Included studies varied in methodological quality with sampling strategies and the representativeness of the study population described in only 21/70 studies (Supplementary Table 2). Most studies (55/57) defined the threshold for white coat or masked hypertension (where appropriate) and examined the home-clinic blood pressure difference as the primary focus of the study (68/70). Forty-six studies identified important confounding variables and 44 of these corrected for this confounding in their analysis. Full details of the multivariate analysis conducted in each study are given in Supplementary Table 3).

**Figure 2.**
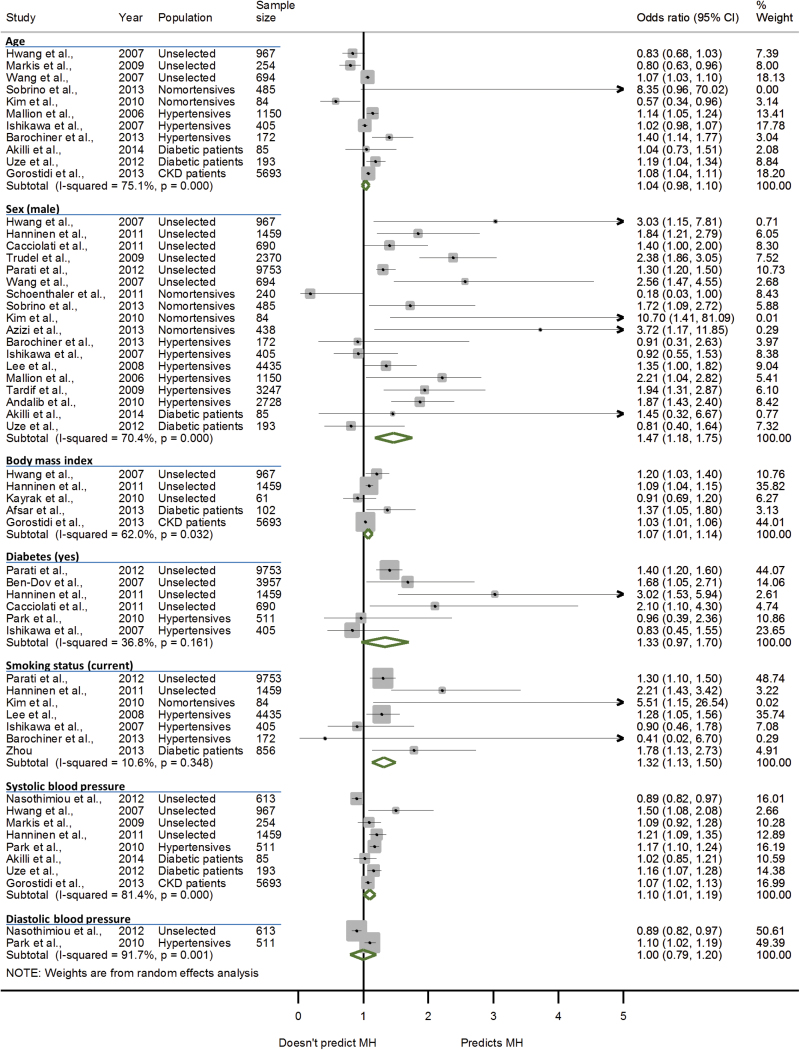
Forest-plot showing pooled odds ratio estimates for the 7 most commonly cited predictors of masked hypertension. Abbreviations: MH, masked hypertension; CKD, chronic kidney disease. Binary predictors were defined using Female sex, no diabetes, and nonsmoker as the reference values (respectively). Continuous predictors were defined as increases in age per 10 years, BMI per 1kg/m^2^ and systolic/diastolic blood pressure per 1mm Hg.

**Figure 3.**
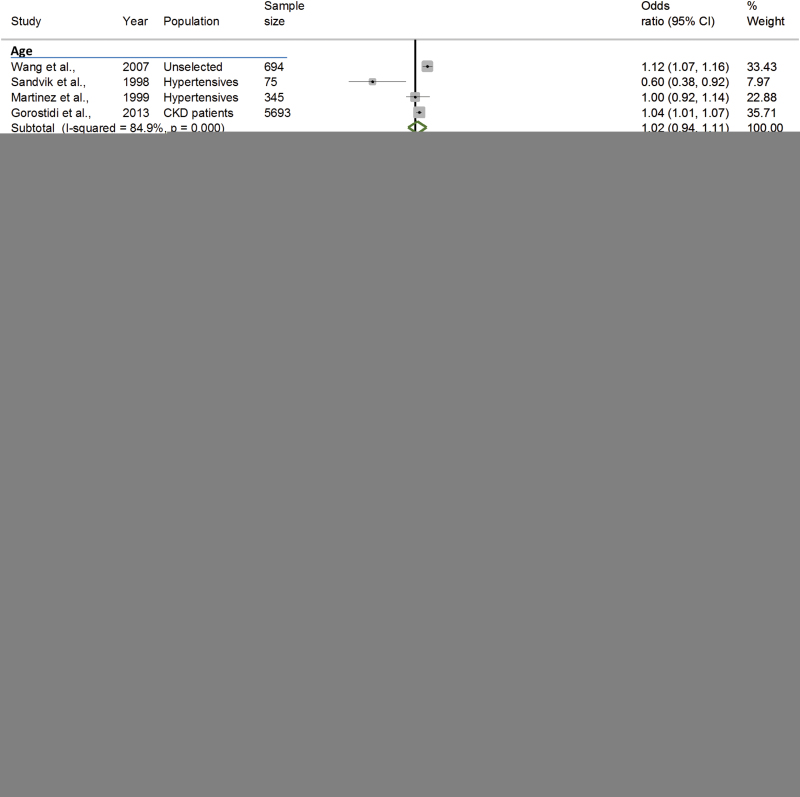
Forest-plot showing pooled odds ratio estimates for the 7 most commonly cited predictors of white coat hypertension. WCH, white coat hypertension; CKD, chronic kidney disease. Binary predictors were defined using female sex, no diabetes, and nonsmoker as the reference values (respectively). Continuous predictors were defined as increases in age per 10 years, BMI per 1kg/m^2^, and systolic/diastolic blood pressure per 1mm Hg.

Included studies reported a total of 60 significant predictors of the home-clinic blood pressure difference, white coat or masked hypertension. The most commonly cited predictors of the home-clinic blood pressure difference were sex (14 studies), age (11 studies), body mass index (BMI, 7 studies), and systolic (12 studies) and diastolic blood pressure (5 studies) (Supplementary Table 4). These factors were also commonly cited as predictors of both white coat and masked hypertension with the addition of diabetes and smoking status (Tables 3 and 4). The overall association between these factors and white coat or masked hypertension was established by pooling ORs for each predictor from 31 studies in a random-effects meta-analysis. Male sex (OR 1.47, 95% CI 1.18–1.75), increasing BMI (per kg/m^2^ increase, OR 1.07, 95% CI 1.01–1.14), current smoking status (OR 1.32, 95% CI 1.13–1.50), and systolic clinic blood pressure (per 1mm Hg increase, OR 1.10, 95% CI 1.01–1.19) were all found to be significant predictors of masked hypertension (Figure 2). Male sex was found to be predictive of not having white coat hypertension (OR 0.57, 95% CI 0.42–0.72) (Figure 3): analyzed with male sex as the reference, female sex was a significant predictor of white coat hypertension (OR 3.38, 95% CI 1.64–6.96). The heterogeneity between studies for sex (*I* ^2^ = 70.4% (masked hypertension); *I* ^2^ = 75.7% (white coat hypertension)), BMI (*I* ^2^ = 62.0%), and systolic blood pressure (*I* ^2^ = 81.4%) predictors of white coat and masked hypertension was significant (*P* < 0.05).

**Table 3.** Predictors of masked hypertension reported in included studies (*n* = 34)

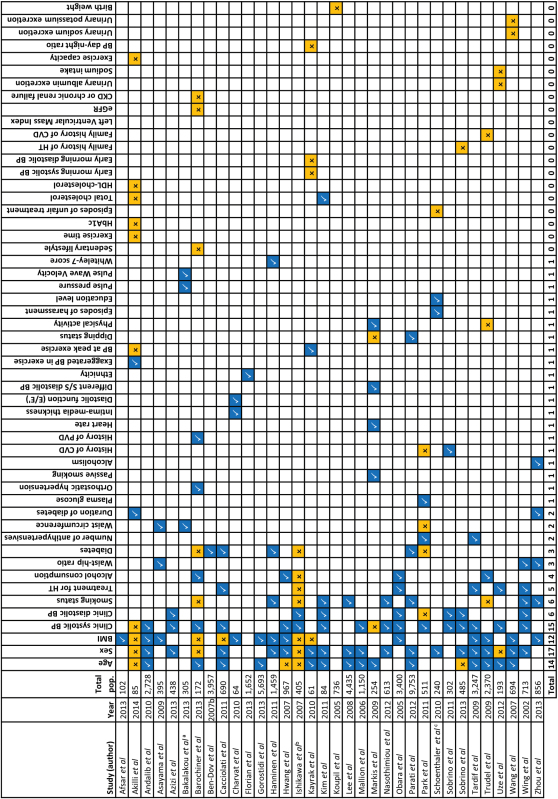

Last row indicates total number of studies citing each factor as a significant predictor of masked hypertension. References mentioned in the table are found in the Supplementary Appendix.

Abbreviations: CVD, cardiovascular disease; PVD, peripheral vascular disease; BP, blood pressure; eGFR, estimated glomerular filtration rate; CKD, chronic kidney disease; HT, hypertension; BMI, body mass index.

^a^Examined masked nocturnal hypertension as the outcome. ^b^Examined masked morning hypertension as the outcome. ^c^Examined “marked” masked hypertension as the outcome.

Significant predictor.

Nonsignificant predictor.

Significant predictor defined as an OR or *ß* coefficient with an associated *P* value of <0.05.

**Table 4.** Predictors of white coat hypertension reported in included studies (*n* = 18)

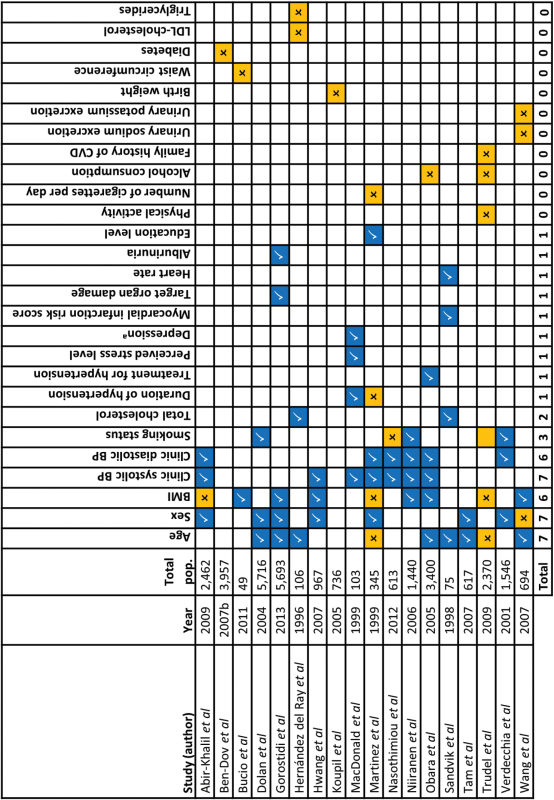

Last row indicates total number of studies citing each factor as a significant predictor of masked hypertension. References mentioned in the table are found in the Supplementary Appendix.

Abbreviations: CVD, cardiovascular disease; BP, blood pressure; BMI, body mass index.

^a^Examined using the Centre for Epidemiological Studies Depression Scale.

Significant predictor.

Nonsignificant predictor.

Significant predictor defined as an OR or *ß* coefficient with an associated *P* value of <0.05.

### Sensitivity analysis

Inclusion of only those studies that used ambulatory blood pressure to define masked hypertension resulted in diabetes becoming a significant predictor (OR 1.42, 95% CI 1.22–1.61) but BMI and systolic blood pressure no longer being predictive. When only studies that used home blood pressure to define masked hypertension were included, only sex remained a significant predictor, although there were insufficient studies to examine the relationship between BMI and masked hypertension. Using ambulatory blood pressure or home blood pressure to define white coat hypertension had no impact on the findings of the primary analysis although there were no longer sufficient data to examine the association with diabetes, smoking status and diastolic blood pressure (studies using ambulatory blood pressure), or age, BMI, and systolic and diastolic blood pressure (studies using home blood pressure). Similar findings were observed in the sensitivity analysis excluding low quality studies that did not account for confounding variables.

In an unselected population, male sex and diabetes were predictive of masked hypertension (OR 1.76, 95% CI 1.29–2.24 (sex); OR 1.48, 95% CI 1.22–1.70 (diabetes)), while in hypertensive patients, only male sex remained significant (OR 1.52, 95% CI 1.11–1.93) for masked uncontrolled hypertension, although there were no longer sufficient data to examine the association with systolic and diastolic blood pressure. Examining only patients from an unselected population, male sex was predictive of not having white coat hypertension (OR 0.47, 95% CI 0.33–0.61) and systolic blood pressure was predictive of having white coat hypertension (OR 1.06, 95% CI 1.04–1.08). In hypertensive patients, male sex remained predictive of not having white coat hypertension (OR 0.62, 95% CI 0.48–0.76), although again, insufficient data were available to examine associations with BMI and systolic or diastolic blood pressure. The observed heterogeneity was not reduced in any sensitivity analyses examining studies by outcome measurement, sample populations, or methodological quality.

## DISCUSSION

This study has systematically reviewed all existing literature evaluating the association between patient characteristics and the home-clinic blood pressure difference. A large number of studies were identified examining a number of common factors which predict the home-clinic blood pressure difference or white coat or masked hypertension. Meta-analyses of the most commonly cited predictors revealed that sex, BMI, smoking status, and systolic blood pressure level were the most important predictors, although these associations were mediated by the method of out-of-office blood pressure monitoring and the population studied. There is scope to incorporate such predictors into a clinical prediction tool which could be used to identify those patients more likely to display a significant masked or white coat effect and therefore better target the use of out-of-office blood pressure monitoring in routine clinical practice.

### Strengths and limitations

This is the largest systematic review to date of studies examining the association between patient factors and the home-clinic blood pressure difference. An extensive search strategy was used in multiple research literature databases to comprehensively capture all published articles relating to the study research question. Not all of the identified studies were directly comparable due to a lack of relevant data or the use of different statistical methods in the original study analyses. Thus, only 31/70 studies could be included in the meta-analysis. While sufficient data were available to analyze the primary outcome of this review, the lower number of studies eligible for meta-analysis meant some sensitivity and subgroup analyses were not possible. For instance, previous studies have suggested that the degree of white coat or masked effect may be affected by attributes of the person taking the clinic blood pressure measurement.^28^ Although an attempt was made to extract details of the person taking clinic blood pressure from each included study, many did not report this or used both doctors and nurses to take readings without distinguishing between the 2, meaning a subgroup analysis by the type of person taking the clinic measurement was not possible.

The methodological quality of studies and the population of study varied widely between included studies and this may have contributed to the observed statistical heterogeneity. Indeed, the significant predictors of masked hypertension changed in sensitivity analyses excluding low quality studies that did not correct for confounding variables, although the statistical heterogeneity between studies remained significant. Only sex remained a significant predictor of both white coat and masked hypertension across patient populations and study quality.

### Comparison with previous literature

A number of previous reviews^20–22^ and clinical guidelines^14^ have discussed possible predictors of white coat and masked hypertension. Indeed, the present review demonstrates that the literature is becoming saturated with studies describing predictors of white coat or masked hypertension. Despite the large volume of articles studying this topic, little insight has been gained over the last 20 years and the patient factors commonly cited as significant predictors of the home-clinic blood pressure difference remain the same: age, sex, BMI, smoking status, and clinic blood pressure level.

Recent studies have examined the influence of patient ethnicity on the home-clinic blood pressure difference. Martin *et al.,* ^29^ studied 770 individuals of White British, South Asian, or African-Caribbean ethnicity and found that when clinic blood pressure was defined using a single reading, non-hypertensive South Asian or African-Caribbean patients displayed less of a home-clinic blood pressure difference compared to White British patients. In contrast, hypertensive patients of South Asian or African-Caribbean origin had a greater home-clinic difference. The present review found only 2 studies examining ethnicity as a predictor of the home-clinic blood pressure difference^30,31^ and neither could be included in the meta-analysis. However, the recent Jackson Heart study^32^ (published after the searches in the present study were conducted) examined a population of 972 African-Americans and found male sex, current smoking status, diabetes, prescribed medication, and clinic blood pressure were significant predictors of masked hypertension. These findings are similar to those of the present review and suggest that our findings may be applicable to some ethnic minority groups.

This is the first systematic review to summarize all available evidence and present pooled estimates describing the most important predictors of white coat and masked hypertension. Seventy studies fulfilled our strict inclusion criteria and 60 different predictors of the home-clinic blood pressure difference were identified. It is unclear from the data included in this review as to why certain factors predict a white coat or masked effect to a greater degree than others. However, it is of interest that, in our analysis, significant predictors appeared to be related to the underlying cardiovascular disease risk associated with each condition: masked hypertension (associated with high cardiovascular disease risk)^18,19^ was more common in patients with characteristics associated with increased cardiovascular risk such as male sex, current smoking status, increasing BMI, and increasing blood pressure.^33,34^ White coat hypertension (associated with lower cardiovascular disease risk)^18,19^ was associated with female sex, which is also associated with lower cardiovascular disease risk (compared to male sex).^33,34^

### Implications for clinical practice

It is important to identify patients with white coat and masked hypertension because failure to do so can result in significant misdiagnosis and mismanagement of hypertension.^35^ Those with white coat hypertension may be prescribed therapy when they do not need it while patients with masked hypertension are likely to be denied potentially beneficial treatment.^15^ Despite the large number of studies citing predictors of white coat and masked hypertension identified in this review, few have proposed a practical method for screening patients in routine clinical practice.^21^ Indeed, screening for white coat or masked hypertension is only useful if it reduces the number of patients potentially eligible for out-of-office monitoring. The number of predictive factors identified in this review makes their use to guide targeting of out-of-office monitoring impractical because a significant proportion of patients attending routine clinical practice are likely to present with at least one of these characteristics.

Some previous studies have suggested methods for targeted use of ABPM, mostly suggesting specific clinic blood pressure thresholds to target monitoring.^36,37^ Viera *et al.* ^38^ examined optimal clinic blood pressure levels for referral for ambulatory monitoring in patients with normal clinic pressure for detection of masked hypertension. They identified a threshold of greater than 120/82mm Hg as optimal but concluded that using clinic blood pressure alone was not an effective method of triaging for out-of-office monitoring because of high referral rates and moderate specificity. They suggested that a combination of factors, perhaps such as those identified in the present review, might be more effective at targeting ABPM efficiently.

The European Society of Hypertension^14^ suggests that practicing physicians consider screening for masked hypertension in high risk patients with normal clinic blood pressure, or screening for white coat hypertension in low risk patients with raised clinic blood pressure. This is still likely to result in a large number of patients being indicated for out-of-office blood pressure monitoring and future work should therefore focus on developing a single, practical, decision aid for targeted screening of white coat or masked hypertension, incorporating all of the significant predictors identified in this review.

There are a number of common patient characteristics that predict the home-clinic blood pressure difference including sex, current smoking status, increasing BMI, and increasing systolic blood pressure. There is scope to incorporate such predictors into a clinical prediction tool which could be used to identify those patients displaying a significant masked or white coat effect in routine clinical practice. Identification of such patients could help to better target antihypertensive treatment at those people with the most to gain.

## DISCLOSURE

R.J.M. has received research funding from Omron and Lloyds Pharmacies in terms of blood pressure monitoring equipment. All other authors declared no conflict of interest.

## Supplementary Material

Supplementary Data
