# Supplementary material for: Predictors of the Home-Clinic Blood Pressure Difference: A Systematic Review and Meta-Analysis
Source: Am J Hypertens. 2015 Sep 22;29(5):614–25. doi: 10.1093/ajh/hpv157 (PMC4829055; doi:10.1093/ajh/hpv157)
Supplement: Supplementary Data [file supp_29_5_614__index.html]

Predictors of the Home-Clinic Blood Pressure Difference: A Systematic Review and Meta-Analysis — Predictors of the Home-Clinic Blood Pressure Difference: A Systematic Review and Meta-Analysis — Supplementary Data 

# Predictors of the Home-Clinic Blood Pressure Difference: A Systematic Review and Meta-Analysis

## Supplementary Data

Data files

- Supplementary Data - Supplementary Data
