# Supplementary material for: Predictors of the Home-Clinic Blood Pressure Difference: A Systematic Review and Meta-Analysis
Source: Am J Hypertens. 2015 Sep 22;29(5):614–25. doi: 10.1093/ajh/hpv157 (PMC4829055; doi:10.1093/ajh/hpv157)
Supplement: Supplementary Data [file supp_hpv157_Home_clinic_diff_SR_online_appendix__20_07_15__FINAL.docx]

**Online appendix**

1. **Study protocol**
2. **eTable 1.** Search strategy designed and used in Ovid MEDLINE®
3. **Data extraction form**
4. **eTable 2.** Assessment of methodological quality
5. **eTable 3.** Statistical modelling and confounding factors examined in each study included in the meta-analysis
6. **eTable 4.** Predictors of the home-clinic blood pressure difference
7. **References**

**Predictors of the home-clinic blood pressure difference: a systematic review (protocol)**

**Background**

Hypertension is an important risk factor for cardiovascular disease,[^1^](#_ENREF_1) the major cause of morbidity and mortality worldwide.[^2^](#_ENREF_2) Effective diagnosis and management depends on accurate measurement of blood pressure which in turn facilitates appropriate targeting of antihypertensive treatment. Ambulatory blood pressure monitoring (ABPM) is considered to be the “gold standard” measure of blood pressure, because multiple readings are taken and because it correlates better with a range of cardiovascular outcomes and end organ damage.[^3-7^](#_ENREF_3) ABPM is usually lower than clinic blood pressure[^8-11^](#_ENREF_8) due to the white coat effect,[^12^](#_ENREF_12) and as such, clinical guidelines recommend that ABPM (or home) blood pressure targets are 5mmHg lower than the corresponding clinic values.[^13^](#_ENREF_13)^,^ [^14^](#_ENREF_14)

But not all patients have lower ABPM (or home) blood pressure than clinic readings. Some display a masked effect, where blood pressures measured with home or ABPM are higher than would be expected for the corresponding clinic blood pressure.[^15^](#_ENREF_15) These patients are often undertreated, leading to increased target organ damage[^16^](#_ENREF_16)^,^ [^17^](#_ENREF_17) and cardiovascular mortality.[^18^](#_ENREF_18)^,^ [^19^](#_ENREF_19) Patients with a significant masked effect could benefit from treatment, but generally do not receive it.[^15^](#_ENREF_15) Conversely, those with a significant white coat effect may be prescribed therapy when they do not need it. Even in the UK, where guidelines[^13^](#_ENREF_13) now recommend that patients with raised clinic blood pressure (>140/90 mmHg) are sent for ABPM to confirm a diagnosis of hypertension, some patients will be sent for uneccessary ABPM.

There are a number of studies proposing factors which predict whether a patient is likely to display a white coat or masked effect.[^20^](#_ENREF_20)^,^ [^21^](#_ENREF_21) However, there is little consensus on which factors are most important or how they should be used in clinical practice to guide diagnosis and management decisions. The present study aims to establish the most important predictors of a significant home-clinic blood pressure difference to inform interventions that can facilitate more accurate blood pressure measurement in routine clinical practice.

**Research questions**

- What factors predict the home-clinic BP difference?
- Which factors have the largest impact on the home-clinic BP difference?
- Does the mean home-clinic BP difference differ between different population types (e.g. hypertensive/normotensive, treated/untreated, etc.)?

**Definitions**

- *Home-clinic blood pressure difference* = difference between blood pressure measured at home (self monitored) or with ABPM and blood pressure measured in the clinic
- *White coat effect* (negative home-clinic blood pressure difference) = blood pressure measured at home or with ABPM is lower than the corresponding clinic blood pressure
- *White coat hypertension* (negative home-clinic blood pressure difference) = blood pressure measured at home or with ABPM is <135/85mmHg but the corresponding clinic blood pressure is >140/90mmHg
- *Masked effect* (positive home-clinic blood pressure difference) = blood pressure measured at home or with ABPM is higher than the corresponding clinic blood pressure
- *Masked hypertension* (positive home-clinic blood pressure difference) = blood pressure measured at home or with ABPM is >135/85mmHg but the corresponding clinic blood pressure is <140/90mmHg

**Design**

This study will systematically review all existing literature examining factors which predict the home-clinic blood pressure difference. Mean values for home-clinic blood pressure difference and coefficients/odds ratios describing the association between patient characteristics and this difference will be extracted and where possible, entered into a meta-analysis.

**Search strategy**

A scoping search has been carried out to identify background literature and provide an estimate of the volume of literature on the topic. A search strategy designed for use with MEDLINE (see appendix) will be adapted to be run across the following databases:

- CINAHL (EBSCO)
- The Cochrane (Wiley) CENTRAL Register of Controlled Trials
- EMBASE (Ovid)
- MEDLINE (Ovid) and MEDLINE In Process (Ovid)
- Science Citation Index – Expanded & Conference Proceedings Citation Index – Science
- The ZETOC (Mimas) database

In order to capture as broad a range of studies as possible, no language or date limits will be applied to the searches although animal studies, and letters, comments and review articles will be excluded. In addition to searches of electronic databases, reference lists of studies included in the review will be checked to identify any further relevant papers.

**Inclusion criteria**

Studies will be selected for full document screening and data extraction based on the following criteria:

- Measure out of office BP
- Measure clinic BP
- Cross-sectional study
- Examine independent variables routinely available or measurable in a Primary Care clinic setting
- Examine the association between these variables and the home-clinic blood pressure difference (outcome variable).
- Must include primary data

**Exclusion criteria**

The present review aims to identify factors which could be utilised by clinicians in the routine diagnosis and management of hypertension in a Primary Care setting. Therefore, studies will be excluded from data extraction if they study:

- Examine patients in hospital for surgery or treatment for a specialist condition (e.g. hemodialysis, pregnancy)
- Examine assessments made in a non-clinical or pharmacy setting
- Study patients aged <18 years

**Data collection**

Data will be extracted from relevant articles identified in the search strategy by JS and BF. Data relating to the definition of clinic BP, out of office BP and home-clinic difference (white coat effect, white coat hypertension, etc.) and coefficients/odds ratios/relative risks which predict this difference will be extracted. In addition, any information about the sample population will be recorded, including patient demographics, mean clinic/out of office blood pressure, mean population home-clinic difference, diagnosis of white coat or masked hypertension, prescribed medication and history of cardiovascular disease events/risk factors.

**Assessment of methodological quality**

As part of the data extraction, the methodological quality and risk of bias of individual studies will be assessed, and sensitivity analyses completed focusing on the highest quality studies. This quality assessment will cover domains on selection bias, detection bias accuracy of measurement, analysis and confounding using a combination of questions from the QUADAS-2[^22^](#_ENREF_22) and CASP[^23^](#_ENREF_23) checklists for assessment of cohort studies.

**Proposed outcomes measures**

The primary outcome of this review will be to identify the most important risk factors for a significant home-clinic blood pressure difference (white coat and/or masked effect). In addition, this review will explore whether such factors differ between populations and whether different definitions of BP (clinic, home or ABPM) affect the mean home-clinic blood pressure difference.

**Data synthesis/Statistical analysis**

Where possible and appropriate, overall findings will be based on a random effects meta-analysis of relative risks or odds ratios relating patient characteristics to a significant home-clinic blood pressure difference. Where feasible, data analysis will account for population heterogeneity caused by other patient factors such as age, sex, blood pressure variability, white coat, masked or isolated systolic hypertension, prescribed medication and cardiovascular disease risk factors.

**Reporting**

This systematic review will be reported according to MOOSE guidelines[^24^](#_ENREF_24) which offer guidance on appropriate reviewing of observational studies. This will ensure that the reporting is to a high quality evidence-based standard.

| **eTable 1.** Search strategy designed and used in Ovid MEDLINE® |
| --- |
| \| No. \| Search term \| No. of articles \| \| --- \| --- \| --- \| \| 1 \| White Coat Hypertension/ \| 117 \| \| 2 \| (white coat and (hypertens* or blood pressure or bp)).ti,ab. \| 1296 \| \| 3 \| (white coat adj (effect? or response?)).ti,ab. \| 383 \| \| 4 \| (clinic effect and (hypertens* or blood pressure or bp)).ti,ab. \| 0 \| \| 5 \| (office hypertension or clinic hypertension).ti,ab. \| 149 \| \| 6 \| Masked Hypertension/ \| 56 \| \| 7 \| (masked adj3 (hypertens* or blood pressure or bp)).ti,ab. \| 395 \| \| 8 \| (masked adj (effect? or response?)).ti,ab. \| 30 \| \| 9 \| (home effect and (hypertens* or blood pressure or bp)).ti,ab. \| 1 \| \| 10 \| ((home clinic? or home office? or clinic? home or office? home) adj difference?).ti,ab. \| 3 \| \| 11 \| Blood Pressure Determination/ \| 16529 \| \| 12 \| blood pressure monitors/ \| 1952 \| \| 13 \| 11 or 12 \| 17861 \| \| 14 \| Office Visits/ \| 5342 \| \| 15 \| physicians/ or general practitioners/ or physicians, family/ or physicians, primary care/ \| 77099 \| \| 16 \| general practice/ or family practice/ \| 63279 \| \| 17 \| 14 or 15 or 16 \| 140436 \| \| 18 \| 13 and 17 \| 578 \| \| 19 \| Sphygmomanometers/ \| 651 \| \| 20 \| ((clinic? or office or surgery or practice) adj5 (blood pressure or bp or sbp or dbp)).ti,ab. \| 4979 \| \| 21 \| ((clinic? or office or surgery or practice) and (blood pressure or bp or sbp or dbp)).ti. \| 1203 \| \| 22 \| ((physician? or doctor? or clinician? or nurse? or practitioner?) adj5 (blood pressure or bp or sbp or dbp)).ti,ab. \| 1266 \| \| 23 \| ((physician? or doctor? or clinician? or nurse? or practitioner?) and (blood pressure or bp or sbp or dbp)).ti. \| 456 \| \| 24 \| 18 or 19 or 20 or 21 or 22 or 23 \| 7210 \| \| 25 \| Blood Pressure Monitoring, Ambulatory/ \| 6762 \| \| 26 \| ((ambulatory or self* or home or 24hr* or 24 hr* or 24hour* or 24 hour*) adj5 (blood pressure or bp or sbp or dbp)).ti,ab. \| 12174 \| \| 27 \| ((ambulatory or self* or home or 24hr* or 24 hr* or 24hour* or 24 hour*) and (blood pressure or bp or sbp or dbp)).ti. \| 4753 \| \| 28 \| (abpm or hbpm or sbpm).ti,ab. \| 1880 \| \| 29 \| 25 or 26 or 27 or 28 \| 14227 \| \| 30 \| 24 and 29 \| 3308 \| \| 31 \| differ*.ti,ab. \| 4187273 \| \| 32 \| correlat*.ti,ab. \| 1244729 \| \| 33 \| compar*.ti,ab. \| 3500076 \| \| 34 \| drop?.ti,ab. \| 63703 \| \| 35 \| linear regression.ti,ab. \| 50344 \| \| 36 \| Reference Values/ \| 143255 \| \| 37 \| 31 or 32 or 33 or 34 or 35 or 36 \| 6917776 \| \| 38 \| 30 and 37 \| 2350 \| \| 39 \| 1 or 2 or 3 or 4 or 5 or 6 or 7 or 8 or 9 or 10 or 38 \| 3457 \| \| 40 \| Prognosis/ \| 351977 \| \| 41 \| risk/ or risk factors/ or risk assessment/ \| 761407 \| \| 42 \| predict*.ti,ab. \| 917385 \| \| 43 \| detect*.ti,ab. \| 1604240 \| \| 44 \| prognos*.ti,ab. \| 360965 \| \| 45 \| risk*.ti,ab. \| 1286487 \| \| 46 \| estimat*.ti,ab. \| 676775 \| \| 47 \| factor?.ti,ab. \| 2152219 \| \| 48 \| determinant?.ti,ab. \| 162234 \| \| 49 \| association?.ti,ab. \| 820306 \| \| 50 \| 40 or 41 or 42 or 43 or 44 or 45 or 46 or 47 or 48 or 49 \| 6096823 \| \| 51 \| 39 and 50 \| 2045 \| \| 52 \| (comment or letter or "review").pt. \| 2905504 \| \| 53 \| 51 not 52 \| 1665 \| |

### Data Extraction Sheet

| Data extracted by |  |
| --- | --- |
| Date of review |  |
| Author |  |
| Publication year |  |

### Paper Details

### Recruitment and setting

| Country of origin | |  |
| --- | --- | --- |
| Recruitment method | |  |
| Setting | Primary care |  |
|  | Outpatient clinic |  |
|  | Community |  |
|  | Other |  |
| Inclusion/exclusion criteria  (list everything) Initiation of BP treatment or continuation? | |  |
| Numbers of participants screened and numbers agreeing to take part | |  |

### Participants at baseline

| Total population |  |
| --- | --- |
| Gender (% female) |  |
| Age (years) |  |
| Office systolic blood pressure (mean) |  |
| Office systolic blood pressure (SD) |  |
| Office diastolic blood pressure (mean) |  |
| Office diastolic blood pressure (SD) |  |
| Ethnicity (% white) |  |
| Hypertensive status at baseline (normotensive/hypertensive based on office readings) |  |
| Prescribed antihypertensive therapy (%) |  |
| History of CVD (yes/no) |  |
| Diabetes (yes/no) |  |
| CKD (yes/no) |  |
| Smoking status (current/non) |  |
| Other co-morbidities |  |
| Other/notes |  |

### Methodological quality of the study (Derived from QUADAS-2 and the CASP checklist for cohort studies)

| **Domain** | **Specific question** | **Answer** (yes, no, or unclear) | **Detail** (reason for decision – quote if possible) |
| --- | --- | --- | --- |
| Patient selection | Was selection of patients appropriate? (e.g. consecutive or random sample) |  |  |
|  | Did the study avoid inappropriate exclusions? (e.g. exclusion according to age – without justification) |  |  |
|  | Was the study sample representative? (e.g. of the intended/reported population of study) |  |  |
| Outcome  measurement | Is the outcome variable measured appropriately? (e.g. Home-clinic BP difference estimated from clinic and ABPM/home readings) |  |  |
|  | If a threshold was used, was it pre-specified? (e.g. definition of white-coat or masked hypertension) |  |  |
| Analysis | Are statistical analyses clearly described? |  |  |
|  | Was the home-clinic difference the primary focus of the study? |  |  |
| Confounding | Were all important confounding factors identified? (e.g. did consider multiple or just a single predictor of the home-clinic BP difference?) |  |  |
|  | Were confounding factors taken into account in the analysis? |  |  |

### Details of Blood pressure measurement

| **Measurement setting** | **Detail** | **Answer** |
| --- | --- | --- |
| Office BP | Number of readings |  |
|  | Interval between readings |  |
|  | Time of day (used in analysis) |  |
|  | Protocol for measurement (e.g. mean of 3, discard 1^st^ reading, etc.) |  |
|  | Monitor type |  |
|  | Person taking measurement |  |
| Home BP | Number of readings |  |
|  | Interval between readings |  |
|  | Time of day (used in analysis) |  |
|  | Protocol for measurement (e.g. mean of 3, discard 1^st^ reading, etc.) |  |
|  | Monitor type |  |
| ABPM | Number of readings |  |
|  | Interval between readings |  |
|  | Number of hours |  |
|  | Time of day (used in analysis; e.g. daytime, nighttime or 24hr) |  |
|  | Monitor type |  |

### Analysis

| Analysis used |  |
| --- | --- |
| Predictor selection (e.g. backward stepwise, etc.) |  |
| Significance for predictor inclusion (p value) |  |
| Predictors considered in model |  |
| Outcome variable |  |
| Definition of outcome variable |  |

### Results

| **Mean blood pressure** | |
| --- | --- |
| Office SBP (mean) (mmHg) |  |
| Office SBP SD (mmHg) |  |
| Office DBP (mean) (mmHg) |  |
| Office DBP SD (mmHg) |  |
| Home SBP (mean) (mmHg) |  |
| Home SBP (SD) (mmHg) |  |
| Home DBP (mean) (mmHg) |  |
| Home DBP (SD) (mmHg) |  |
| Daytime ABPM SBP (mean) (mmHg) |  |
| Daytime ABPM SBP (SD) (mmHg) |  |
| Daytime ABPM DBP (mean) (mmHg) |  |
| Daytime ABPM DBP (SD) (mmHg) |  |
| 24hr ABPM SBP (mean) (mmHg) |  |
| 24hr ABPM SBP (SD) (mmHg) |  |
| 24hr ABPM DBP (mean) (mmHg) |  |
| 24hr ABPM DBP (SD) (mmHg) |  |
| Home-clinic SBP difference (mean) |  |
| Home-clinic DBP difference (SD) |  |
| Home-clinic SBP difference (mean) |  |
| Home-clinic DBP difference (SD) |  |

| **Significant predictors of home-clinic difference (after adjustment for confounding)** | | | |
| --- | --- | --- | --- |
| **Predictor** | **Odds ratio** | **95% CI lower** | **95% CI upper** |
|  |  |  |  |
|  |  |  |  |
|  |  |  |  |
|  |  |  |  |
|  |  |  |  |
|  |  |  |  |
|  |  |  |  |
|  |  |  |  |
|  |  |  |  |
|  |  |  |  |
|  |  |  |  |
|  |  |  |  |
|  |  |  |  |
|  |  |  |  |

### Notes

|  |
| --- |

**eTable 2.** Assessment of methodological quality

| Study | Year | Sampling appropriate? | Inappropriate exclusions avoided? | Representative population? | Outcome measured appropriately? | Outcome threshold defined? | Analysis clearly described? | Home-clinic diff primary focus of the study? | Important confounding identified? | Confounding taken into account? |
| --- | --- | --- | --- | --- | --- | --- | --- | --- | --- | --- |
| Abir-Khalil et al.,[^25^](#_ENREF_25) | 2009 | Yes | Yes | Unclear | Yes | Yes | No | yes | Unclear | Yes |
| Afsar et al.,[^26^](#_ENREF_26) | 2013 | Unclear | Yes | Unclear | Yes | Yes | No | Yes | yes | Yes |
| Akilli et al.,[^27^](#_ENREF_27) | 2014 | Yes | Yes | Yes | Yes | Yes | Yes | Yes | Yes | Yes |
| Andalib et al.,[^28^](#_ENREF_28) | 2010 | No | Yes | Unclear | Unclear | Yes | Yes | Yes | Yes | Yes |
| Asayama et al.,[^29^](#_ENREF_29) | 2009 | Yes | No | No | Yes | Yes | No | Yes | yes | Yes |
| Azizi et al.,[^30^](#_ENREF_30) | 2013 | Unclear | Unclear | Unclear | Unclear | Yes | No | yes | No | Unclear |
| Bakalakou et al.,[^31^](#_ENREF_31) | 2013 | Unclear | Unclear | Unclear | Unclear | Yes | No | Yes | No | Unclear |
| Barochiner et al.,[^32^](#_ENREF_32) | 2013 | Yes | Yes | Unclear | Yes | Yes | Yes | yes | yes | Yes |
| Ben-Dov et al.,[^33^](#_ENREF_33) | 2007a | Yes | Yes | Yes | Yes | Yes | No | Yes | Yes | Yes |
| Ben-Dov et al.,[^34^](#_ENREF_34) | 2007b | Yes | Unclear | Yes | Yes | Yes | Yes | Yes | Yes | Yes |
| Bucio et al.,[^35^](#_ENREF_35) | 2011 | Unclear | Unclear | Unclear | Unclear | No | No | yes | No | No |
| Cacciolati et al.,[^36^](#_ENREF_36) | 2011 | Yes | No | No | Yes | Yes | Yes | Yes | Yes | Yes |
| Calvo-Vargas et al.,[^37^](#_ENREF_37) | 1999 | Unclear | Unclear | Unclear | Unclear | Yes | No | yes | Unclear | Unclear |
| Charvat et al.,[^38^](#_ENREF_38) | 2010 | Unclear | Unclear | Unclear | Unclear | Yes | No | Yes | Unclear | Unclear |
| Dolan et al.,[^39^](#_ENREF_39) | 2004 | Yes | Unclear | Unclear | Yes | Yes | Yes | Yes | Yes | Yes |
| Florian et al.,[^40^](#_ENREF_40) | 2013 | Yes | Unclear | Unclear | Unclear | Yes | No | Yes | No | Yes |
| Gorostidi et al.,[^41^](#_ENREF_41) | 2013 | Yes | Yes | Unclear | Yes | Yes | Yes | Yes | yes | Yes |
| Gualdiero et al.,[^42^](#_ENREF_42) | 2000 | Yes | Yes | Yes | Yes | - | No | Yes | Yes | Yes |
| Hanninen et al.,[^43^](#_ENREF_43) | 2011 | Yes | Yes | No | Yes | Yes | Yes | yes | No | Yes |
| Hermida et al.,[^44^](#_ENREF_44) | 2004 | Unclear | Unclear | Unclear | Yes | Yes | No | Yes | yes | Yes |
| Hernández del Ray[^45^](#_ENREF_45) | 1996 | Yes | Yes | Yes | No | Yes | Yes | Yes | Yes | Yes |
| Hiraizumi et al.,[^46^](#_ENREF_46) | 1998 | Unclear | Unclear | Unclear | Yes | Yes | No | Yes | No | Unclear |
| Horikawa et al.,[^47^](#_ENREF_47) | 2008 | Unclear | Unclear | Unclear | Yes | - | No | Yes | Yes | Yes |
| Hozawa et al., [^48^](#_ENREF_48) | 2001 | Yes | No | Yes | Yes | - | No | Yes | No | Yes |
| Huang et al., [^49^](#_ENREF_49) | 2010 | Yes | Yes | Yes | Yes | Yes | No | Yes | Yes | Yes |
| Hwang et al., [^50^](#_ENREF_50) | 2007 | Yes | Yes | Unclear | Yes | Yes | No | Yes | Yes* | Yes |
| Iimuro et al.,[^51^](#_ENREF_51) | 2013 | Yes | Yes | Yes | Yes | Yes | Yes | Yes | yes | Yes |
| Ishikawa et al.,[^52^](#_ENREF_52) | 2007 | Unclear | Yes | Unclear | Yes | Yes | No | Yes | Yes | Yes |
| Jhalani et al.,[^53^](#_ENREF_53) | 2005 | Unclear | Yes | Unclear | Yes | - | No | yes | Yes | Yes |
| Kabutoya et al.,[^54^](#_ENREF_54) | 2010 | Unclear | Yes | Unclear | Yes | - | No | Yes | Yes | Yes |
| Kayrak et al.,[^55^](#_ENREF_55) | 2010 | Unclear | No | Yes | Yes | Yes | Yes | Yes | Yes | Yes |
| Kim et al.,[^56^](#_ENREF_56) | 2011 | Yes | Yes | Unclear | Yes | Yes | No | Yes | Yes | Yes |
| Koupil et al.,[^57^](#_ENREF_57) | 2005 | Yes | No | Yes | Yes | Yes | No | No | No | Yes |
| Labinson et al.,[^58^](#_ENREF_58) | 2008 | Unclear | Yes | Unclear | Yes | - | Yes | Yes | Yes | Yes |
| Lee et al.,[^59^](#_ENREF_59) | 2008 | Yes | No | Unclear | Yes | Yes | no | No | Yes | Yes |
| Lerman et al.,[^60^](#_ENREF_60) | 1989 | Unclear | Yes | Unclear | Yes | Yes | No | yes | No | Yes |
| Lindbaek et al.,[^61^](#_ENREF_61) | 2003 | Unclear | Yes | Unclear | Yes | Yes | Yes | Yes | No | Yes |
| MacDonald et al.,[^62^](#_ENREF_62) | 1999 | Yes | Yes | Yes | Yes | Yes | No | Yes | No | Yes |
| Mallion et al.,[^63^](#_ENREF_63) | 2006 | Unclear | Yes | Unclear | Yes | Yes | Yes | Yes | Yes | Yes |
| Manios et al.,[^64^](#_ENREF_64) | 2008 | Yes | Yes | Unclear | Yes | Yes | Yes | Yes | Yes | Yes |
| Mansoor et al.,[^65^](#_ENREF_65) | 1996 | Yes | Yes | Yes | Yes | - | Yes | Yes | Yes | Yes |
| Markis et al.,[^66^](#_ENREF_66) | 2009 | Yes | Yes | Yes | Yes | Yes | Yes | Yes | Yes | Yes |
| Martinez et al.,[^67^](#_ENREF_67) | 1999 | Yes | Unclear | Yes | Yes | Yes | Yes | Yes | Yes | Yes |
| Nasothimiou et al.,[^68^](#_ENREF_68) | 2012 | Yes | No | Unclear | Yes | Yes | No | Yes | yes | Yes |
| Niiranen et al.,[^69^](#_ENREF_69) | 2006 | Yes | No | No | Yes | Yes | Yes | Yes | Yes | Yes |
| Obara et al.,[^70^](#_ENREF_70) | 2005 | Unclear | Unclear | Unclear | Yes | Yes | No | Yes | Unclear | Unclear |
| Parati et al.,[^71^](#_ENREF_71) | 2012 | Unclear | Unclear | Unclear | Unclear | Yes | No | Yes | Yes | Yes |
| Park et al.,[^72^](#_ENREF_72) | 2011 | Yes | Yes | Yes | Yes | Yes | Yes | yes | No | Yes |
| Rassmussen et al.,[^73^](#_ENREF_73) | 1998 | Yes | Yes | Unclear | Yes | Yes | Yes | Yes | Yes | Yes |
| Rodrigues et al.,[^74^](#_ENREF_74) | 2009 | Unclear | Unclear | Unclear | Unclear | - | No | yes | Unclear | Unclear |
| Sandvik et al.,[^75^](#_ENREF_75) | 1998 | Yes | Yes | Yes | Unclear | Yes | No | Yes | No | Yes |
| Schoenthaler et al.,[^76^](#_ENREF_76) | 2010 | Unclear | No | Yes | Yes | Yes | No | Yes | Yes | Yes |
| Sheppard et al.,[^77^](#_ENREF_77) | 2014 | Unclear | Yes | Yes | Yes | Yes | No | Yes | No | No |
| Smirnova et al.,[^78^](#_ENREF_78) | 2009 | Unclear | No | Unclear | Unclear | No | No | Yes | Yes | Yes |
| Sobrino et al.,[^79^](#_ENREF_79) | 2013 | Yes | Yes | Unclear | Yes | Yes | Yes | Yes | Yes | Yes |
| Sobrino et al.,[^80^](#_ENREF_80) | 2011 | Yes | Yes | Unclear | Yes | Yes | Yes | Yes | Yes | Unclear |
| Spruill et al.,[^81^](#_ENREF_81) | 2007 | Unclear | No | Unclear | Yes | - | No | Yes | Yes | Yes |
| Streitel et al.,[^82^](#_ENREF_82) | 2011 | Unclear | Yes | Unclear | Yes | - | No | Yes | Yes | Yes |
| Sung et al.,[^83^](#_ENREF_83) | 2013 | Unclear | Yes | Yes | Yes | Yes | No | Yes | Yes | Yes |
| Tam et al.,[^84^](#_ENREF_84) | 2007 | Yes | No | Unclear | Unclear | Yes | No | Yes | Yes | Yes |
| Tardif et al.,[^85^](#_ENREF_85) | 2009 | Unclear | Unclear | Unclear | Unclear | Yes | No | Yes | yes | Unclear |
| Thomas et al.,[^86^](#_ENREF_86) | 2012 | Unclear | No | Unclear | Unclear | - | No | Yes | Unclear | Unclear |
| Trudel et al.,[^87^](#_ENREF_87) | 2009 | Yes | Yes | Yes | Unclear | Yes | Yes | Yes | yes | Yes |
| Tsai et al.,[^88^](#_ENREF_88) | 2003 | Unclear | Unclear | Unclear | Yes | - | No | Yes | No | Yes |
| Uze et al.,[^89^](#_ENREF_89) | 2012 | Unclear | Yes | Unclear | Yes | Yes | Yes | Yes | Yes | Yes |
| Verdecchia et al.,[^90^](#_ENREF_90) | 2001 | Unclear | Yes | Unclear | Yes | Yes | No | Yes | No | Yes |
| Wang et al.,[^91^](#_ENREF_91) | 2007 | Yes | Yes | No | Yes | Yes | Yes | Yes | yes | Yes |
| Wing et al.,[^92^](#_ENREF_92) | 2002 | Unclear | Yes | Yes | Yes | Yes | No | Yes | yes | No |
| Yoon et al.,[^93^](#_ENREF_93) | 2012 | Unclear | Yes | Unclear | Yes | - | No | Yes | Unclear | Unclear |
| Zhou et al.,[^94^](#_ENREF_94) | 2013 | Yes | Yes | Unclear | Yes | Yes | No | Yes | No | Yes |

*White coat hypertension model only corrected for sex

**eTable 3.** Statistical modelling and confounding factors examined in each study included in the meta-analysis

| Author | Year | Analysis | Outcome of interest | Predictors modelled |
| --- | --- | --- | --- | --- |
| Abir-Khalil et al., | 2009 | Multivariate logistic regression | White coat hypertension | Sex, BMI, grade of hypertension (+ other factors considered in a univariate analysis but not stated) |
| Afsar et al., | 2013 | Multivariate logistic regression | Masked hypertension | Age, sex, smoking status, BMI, wait circumference, waist-hip ratio, conicity index, average fasting blood glucose, total cholesterol, triglyceride, uric acid, creatinine clearance, 24-hour urine protein excretion rate, and 24-hour urine albumin excretion rate |
| Akilli et al., | 2014 | Multivariate logistic regression | Masked hypertension | Age, sex, BMI, total cholesterol, HbA1C, duration of diabetes (years), office SBP, exercise duration, exercise capacity, EBPR status and SBP at peak exercise. |
| Andalib et al., | 2010 | Multivariatelogistic regression | Masked hypertension | Age, sex, office systolic blood pressure or diastolic blood pressure, BMI, years since diagnosis of hypertension, concomitant medications (calcium channel blocker, beta blocker, angiotensin converting, enzyme inhibitor), chronic heart failure, dyslipidaemia, post-myocardial infarction, left ventricular hypertrophy. |
| Azizi et al., | 2013 | Multivariate logistic regression | Masked hypertension | Unclear - at least sex and clinic BP (prehypertension) |
| Barochiner et al., | 2013 | Multivariate logistic regression | Masked hypertension | Age, sedentary lifestyle, alcohol consumption, eGFR, history of peripheral artery disease, office sitting systolic BP level, sex, smoking habits, body mass index, diabetes + other candidate predictors not described in the analysis |
| Ben-Dov et al., | 2007b | Multivariate logistic regression | White coat and masked hypertension | Age, sex, BMI, treated hypertension, treated diabetes |
| Cacciolati et al., | 2011 | Multivariate logistic regression | Masked hypertension | Age, sex, BMI, office sBP, diabetes and antihypertensive medication |
| Gorostidi et al., | 2013 | Multivariate logistic regression | White coat and masked hypertension | Age, sex, duration of hypertension, body mass index (BMI), waist circumference, eGFR, albuminuria, diabetes, dyslipidemia, target-organ damage, and CVD. |
| Hanninen et al., | 2011 | Multivariate logistic regression | Masked hypertension | Sex, BMI, smoking status, diabetes, hypercholesterolaemia, Whiteley-7 score (hyperchondria) |
| Hwang et al., | 2007 | Multivariate logistic regression | White coat and masked hypertension | Age, sex, BMI, clinic sBP habitual drinking |
| Ishikawa et al., | 2007 | Multivariate logistic regression | Masked (morning) hypertension | Age, sex, BMI, Smoker, drinker, hyperlipidemia, diabetes or impaired glucose tolerance, antihypertensive drug classes, CBP>130/85, CBP <130/85 |
| Kayrak et al., | 2009 | Multivariate logistic regression | Masked hypertension | Age, sex, BMI, WBC, non-HDL cholesterol, total cholesterol/HDL cholesterol ratio, peak exercise DBP, DBP during the third minute of recovery, a decrease in DBP at night, early morning average BPs |
| Kim et al., | 2011 | Multivariate logistic regression | Masked hypertension | Age, sex, clinic BP, total cholesterol and smoking |
| Lee et al., | 2008 | Multivariate logistic regression | Masked hypertension | Age, sex, smoking status, obesity, diabetes mellitus, metabolic syndrome, or family history of cardiovascular disease |
| Mallion et al., | 2006 | Multivariate logistic regression | Masked hypertension | Age, sex, office sBP |
| Markis et al., | 2009 | Multivariate logistic regression | Masked hypertension | Age, sex, BMI, waist circumference, clinic BP, clinic heart rate, Passive smoking (+duration of exposure, intensity of exposure), alcohol consumption, diet, sodium intake, physical activity, coffee consumption, socio-economic status, plasma glucose, LDL cholesterol, eGFR, LVMI, BP dipping status, S/S differences (sBP, dBP, HR). |
| Martinez et al., | 1999 | Multivariate logistic regression | White coat hypertension | Age, sex, BMI, duration of hypertension, educational level, clinic BP and smoking (number of cigarettes per day) |
| Nasothimiou et al., | 2012 | Multivariate logistic regression | White coat and masked hypertension | Age, sex, BMI, systolic/diastolic clinic BP, number of HBP and awake ambulatory BP readings, cardiovascular disease, diabetes, smoking, alcohol intake and treatment for hypertension |
| Niiranen et al., | 2006 | Multivariate logistic regression | White coat hypertension | Age, sex, BMI, smoking status and level of education, clinic systolic and diastolic BP and heart rate, Beck Depression Inventory score and Toronto Alexithymia Scale score |
| Obara et al., | 2005 | Multivariate logistic regression | White coat and masked hypertension | Unclear - at least sex, BMI, clinic sBP, alcohol consumption and on >2 antihypertensives |
| Parati et al., | 2012 | Multivariate logistic regression | Masked hypertension | Age, sex, diabetes, dyslipidemia, antihypertensive treatment, alcohol, smoking, obesity, age, dipper status |
| Park et al., | 2011 | Multivariate logistic regression | Masked hypertension | Waist, fasting blood glucose, Diabetes, Dyslipidemia, Family history of premature CVD, clinic BP, medication type, number of antihypertensives |
| Sandvik et al., | 1998 | Multivariate logistic regression | White coat hypertension | Age, total cholesterol, heart rate, myocardial infarction risk score |
| Schoenthaler et al., | 2010 | Multivariate logistic regression | (Marked) masked hypertension | Age, sex, ethnicity, BMI, education, harassment, unfair treatment |
| Sobrino et al., | 2013 | Multivariate logistic regression | Masked hypertension | Age, sex, profession, clinic BP (prehypertension), BMI, family history of premature CVD or hypertension, smoking, alcohol abuse, dyslipidaemia |
| Tardif et al., | 2009 | Unclear | Masked hypertension | Age, sex, BMI, number of antihypertensives, concomitant use of ß-blockers or ACE inhibitors with valsartan, clinic BP (<135mmHg), congestive heart failure, dyslipidemia, post myocardial infarction status, LVH, years on treatment, concomitant use of hydrochlorathiazide or calcium chanel blockers with valsartan |
| Trudel et al., | 2009 | Polytomic Logistic regression | White coat and masked hypertension | Age, sex, BMI, smoking status, Alcohol consumption, physical activity, family history of CVD |
| Uze et al., | 2012 | Multivariate logistic regression | Masked hypertension | Age, sex, BMI, HbA1c, total cholesterol, eGFR, urinary albumin excretion rate, sodium intake, number of antihypertensives, use of an angiotensin converting enzyme inhibitor or angiotensin receptor blocker, use of a calcium channel blocker, use of a diuretic, office BP |
| Wang et al., | 2007 | Multivariate logistic regression | White coat and masked hypertension | Age, sex, BMI, current smoking, drinking, energy expenditure during physical activity urinary sodium and potassium excretion |
| Zhou et al., | 2013 | Multivariate logistic regression | Masked hypertension | Unclear – at least BMI, waist-height ratio, 6-15 years since diagnosis of type II diabetes, smoking, alcoholism |

BMI = body mass index; BP = blood pressure; CVD = cardiovascular disease; LVMI = left ventricular mass index; LVH = left ventricular hypertrophy; HR = heart rate

**eTable 4.** Predictors of the home-clinic blood pressure difference reported in included studies (n=26)

| **Study (author)** | **Year** | **Total pop.** | **Age** | **Sex** | **BMI** | **Clinic systolic BP** | **Clinic diastolic BP** | **Treatment for hypertension** | **Smoking status** | **Diabetes** | **History of myocardial infarction** | **Family history of CVD** | **BP variability** | **Pulse pressure** | **Left Ventricular Mass Index** | **Heart rate** | **Anxiety** | **Left ventricular hypertrophy** | **Type of Diabetes** | **Duration of hypertension** | **Plasma glucose** | **Orthostatic hypertension** | **Alcohol consumption** | **Heart rate variability** | **Pulse Wave Velocity** | **Amplitude of backward pressure** | **eGFR** | **CKD or chronic renal failure** | **Ethnicity** | **Household income** | **BP day-night ratio** | **Expectancy** | **Anger** | **Depression** | **Maladaption** | **Perceived hypertensive status** | **Clinic systolic BP drop** | **Clinic systolic BP slope** | **Clinic systolic BP quadratic** | **General wellbeing (questionnaire)** | **Negative events in patient life** | **Height** | **HDL-cholesterol** | **Triglycerides** | **Hypercholesterolemia** | **Uric acid level** | **Difficulty sleeping** | **Nocturia (nighttime urination)** | **Season of the year** | **Alanine aminotransferase** | **Aspartate aminotransferase** | **Tension** |
| --- | --- | --- | --- | --- | --- | --- | --- | --- | --- | --- | --- | --- | --- | --- | --- | --- | --- | --- | --- | --- | --- | --- | --- | --- | --- | --- | --- | --- | --- | --- | --- | --- | --- | --- | --- | --- | --- | --- | --- | --- | --- | --- | --- | --- | --- | --- | --- | --- | --- | --- | --- | --- |
| Ben-Dov et al., | 2007a | 3,928 |  |  | **🗸** |  |  |  |  |  |  |  |  |  |  |  |  |  |  |  |  |  |  |  |  |  |  |  |  |  |  |  |  |  |  |  |  |  |  |  |  |  |  |  |  |  |  |  |  |  |  |  |
| Calvo-Vargas et al., | 1999 | 243 | **🗸** | **🗸** | **🗸** |  |  |  |  |  |  |  |  |  |  |  |  |  |  |  |  |  |  |  |  |  |  |  |  |  |  |  |  |  |  |  |  |  |  |  |  |  |  |  |  |  |  |  |  |  |  |  |
| Gualdiero et al., | 2000 | 1,553 | **🗸** | **🗸** | **🗸** |  |  | **🗴** |  |  |  |  |  |  |  |  |  |  |  |  |  |  |  |  |  |  |  |  | **🗴** |  |  |  |  |  |  |  |  |  |  |  |  |  |  |  |  |  |  |  |  |  |  |  |
| Hermida et al., | 2004 | 837 | **🗸** | **🗸** | **🗸** |  |  |  |  |  |  |  |  |  |  |  |  |  |  |  |  |  |  |  |  |  |  |  |  |  | **🗸** |  |  |  |  |  |  |  |  |  |  |  |  |  |  |  |  |  |  |  |  |  |
| Hiraizumi et al., | 1998 | 86 |  |  |  |  |  |  |  |  |  |  |  |  |  |  |  |  |  |  |  |  |  |  |  |  |  |  |  |  |  |  | **🗴** | **🗸** | **🗸** |  |  |  |  |  |  |  |  |  |  |  |  |  |  |  |  | **🗴** |
| Horikawa et al., | 2008 | 3,308 | **🗸** | **🗸** | **🗸** |  |  |  | **🗴** |  | **🗸** | **🗸** |  |  |  |  |  |  |  |  |  | **🗸** | **🗸** |  |  |  |  |  |  |  |  |  |  |  |  |  |  |  |  |  |  |  |  |  |  | **🗴** |  |  |  |  |  |  |
| Hozawa et al., | 2001 | 1,789 | **🗸** |  |  |  |  | **🗸** | **🗸** |  |  |  |  | **🗸** |  |  |  |  |  |  |  |  |  |  |  |  |  |  |  |  |  |  |  |  |  |  |  |  |  |  |  |  |  |  |  |  |  |  |  |  |  |  |
| Huang et al., | 2010 | 121 |  | **🗸** | **🗴** | **🗸** |  |  |  |  |  |  |  |  |  | **🗸** |  |  |  |  |  |  |  |  |  |  |  |  |  |  |  |  |  |  |  |  |  |  |  |  |  |  | **🗴** | **🗴** |  |  |  |  |  | **🗴** | **🗴** |  |
| Iimuro et al., | 2013 | 964 | **🗴** | **🗴** |  |  |  | **🗸** |  | **🗸** |  |  |  |  |  |  |  |  |  |  |  |  |  |  |  |  | **🗴** |  |  |  |  |  |  |  |  |  |  |  |  |  |  |  |  |  |  |  | **🗴** | **🗴** | **🗴** |  |  |  |
| Jhalani et al., | 2005 | 226 | **🗸** | **🗴** |  |  |  |  |  |  |  |  |  |  |  |  | **🗸** |  |  |  |  |  |  |  |  |  |  |  |  |  |  | **🗸** |  |  |  |  |  |  |  |  |  |  |  |  |  |  |  |  |  |  |  |  |
| Kabutoya et al., | 2010 | 969 | **🗸** | **🗴** |  |  |  | **🗸** | **🗴** | **🗴** | **🗸** |  |  |  |  |  |  |  |  |  |  |  |  |  |  |  |  | **🗴** |  |  |  |  |  |  |  |  |  |  |  |  |  |  |  |  |  |  |  |  |  |  |  |  |
| Labinson et al., | 2008 | 65 | **🗴** | **🗴** | **🗸** |  |  | **🗴** | **🗴** |  |  |  |  |  |  |  |  |  |  |  |  |  |  |  |  |  |  |  |  |  |  |  |  |  |  |  |  |  |  |  |  |  |  |  |  |  |  |  |  |  |  |  |
| Lerman et al., | 1989 | 98 | **🗸** |  |  |  |  | **🗸** |  |  |  |  |  |  |  |  |  |  |  |  |  |  |  |  |  |  |  |  |  |  |  |  | **🗸** |  |  |  |  |  |  |  |  |  |  |  |  |  |  |  |  |  |  |  |
| Lindbaek et al., | 2003 | 221 | **🗸** | **🗸** |  | **🗸** | **🗸** | **🗸** | **🗸** |  |  | **🗸** |  |  |  |  |  |  |  |  |  |  |  |  |  |  |  |  |  |  |  |  |  |  |  |  |  |  |  |  |  |  |  |  |  |  |  |  |  |  |  |  |
| Manios et al., | 2008 | 2,004 | **🗸** | **🗸** | **🗴** | **🗸** | **🗸** | **🗸** | **🗸** | **🗴** |  |  | **🗸** |  |  |  |  |  |  |  |  |  |  |  |  |  |  |  |  |  |  |  |  |  |  |  |  |  |  |  |  |  |  |  | **🗴** |  |  |  |  |  |  |  |
| Mansoor et al., | 1996 | 64 | **🗸** |  |  |  |  |  |  |  |  |  |  |  |  |  |  |  |  | **🗸** |  |  |  |  |  |  |  |  |  |  |  |  |  |  |  |  |  |  |  |  |  |  |  |  |  |  |  |  |  |  |  |  |
| Rassmussen et al., | 1998 | 1,855 | **🗸** | **🗸** | **🗸** | **🗸** | **🗸** |  |  | **🗸** |  |  |  |  | **🗸** | **🗸** |  |  |  |  |  |  |  |  | **🗴** |  |  |  |  |  |  |  |  |  |  |  |  |  |  |  |  |  |  |  |  |  |  |  |  |  |  |  |
| Rodrigues et al., | 2009 | 566 | **🗸** | **🗸** | **🗸** | **🗸** | **🗸** |  |  |  |  |  |  |  |  |  |  |  | **🗸** |  |  |  |  |  |  |  |  |  |  |  |  |  |  |  |  |  |  |  |  |  |  |  |  |  |  |  |  |  |  |  |  |  |
| Smirnova et al., | 2009 | 39 | **🗸** | **🗸** | **🗸** | **🗸** | **🗸** |  |  |  |  |  |  |  |  |  |  | **🗸** |  |  |  |  |  |  |  |  |  |  |  |  |  |  |  |  |  |  |  |  |  | **🗴** | **🗴** | **🗴** |  |  |  |  |  |  |  |  |  |  |
| Spruill et al., | 2007 | 214 |  |  |  |  |  |  |  |  |  |  |  |  |  |  | **🗸** |  |  |  |  |  |  |  |  |  |  |  |  |  |  |  |  |  |  | **🗸** |  |  |  |  |  |  |  |  |  |  |  |  |  |  |  |  |
| Streitel et al., | 2011 | 252 | **🗴** | **🗴** | **🗴** |  |  |  |  |  |  |  |  |  |  |  | **🗴** |  |  |  |  |  |  |  |  |  |  |  |  | **🗸** |  |  |  |  |  |  |  |  |  |  |  |  |  |  |  |  |  |  |  |  |  |  |
| Sung et al., | 2013 | 1,257 | **🗸** | **🗸** |  |  |  |  |  |  |  |  | **🗸** |  | **🗸** |  |  |  |  |  | **🗸** |  |  | **🗸** | **🗸** | **🗸** |  |  |  |  |  |  |  |  |  |  |  |  |  |  |  |  |  |  |  |  |  |  |  |  |  |  |
| Thomas et al., | 2012 | 2,381 |  | **🗸** |  | **🗸** |  |  |  |  |  |  |  |  |  |  |  |  |  |  |  |  |  |  |  |  | **🗸** | **🗸** | **🗸** |  |  |  |  |  |  |  |  |  |  |  |  |  |  |  |  |  |  |  |  |  |  |  |
| Tsai et al., | 2003 | 41 |  | **🗸** |  | **🗸** |  |  |  |  |  |  |  |  |  |  |  |  |  |  |  |  |  |  |  |  |  |  |  |  |  |  |  |  |  |  |  |  |  |  |  |  |  |  |  |  |  |  |  |  |  |  |
| Yoon et al., | 2012 | 1,087 |  |  |  |  |  |  |  |  |  |  |  | **🗸** |  |  |  |  |  |  |  |  |  |  |  |  |  |  |  |  |  |  |  |  |  |  |  |  |  |  |  |  |  |  |  |  |  |  |  |  |  |  |
| Sheppard et al., | 2014 | 220 |  |  |  |  |  |  |  |  |  |  |  |  |  |  |  |  |  |  |  |  |  |  |  |  |  |  |  |  |  |  |  |  |  |  | **🗸** | **🗸** | **🗸** |  |  |  |  |  |  |  |  |  |  |  |  |  |
|  |  | **Total** | **15** | **13** | **7** | **8** | **5** | **5** | **3** | **2** | **2** | **2** | **2** | **2** | **2** | **2** | **2** | **1** | **1** | **1** | **1** | **1** | **1** | **1** | **1** | **1** | **1** | **1** | **1** | **1** | **1** | **1** | **1** | **1** | **1** | **1** | **1** | **1** | **1** | **0** | **0** | **0** | **0** | **0** | **0** | **0** | **0** | **0** | **0** | **0** | **0** | **0** |

Last row indicates total number of studies citing each factor as a significant predictor of masked hypertension

CVD=Cardiovascular Disease; BP=blood pressure; BMI=Body mass index; eGFR=estimated Glomerular Filtration Rate; CKD=Chronic Kidney Disease

Significant predictor;

**🗸**

Non-significant predictor

**🗴**

**References**

1. Lewington S, Clarke R, Qizilbash N, Peto R, Collins R. Age-specific relevance of usual blood pressure to vascular mortality: a meta-analysis of individual data for one million adults in 61 prospective studies. *Lancet* 2002; **360**(9349): 1903-13.

2. Ezzati M, Lopez AD, Rodgers A, Vander Hoorn S, Murray CJ. Selected major risk factors and global and regional burden of disease. *Lancet* 2002; **360**(9343): 1347-60.

3. Staessen JA, Thijs L, Fagard R, et al. Predicting cardiovascular risk using conventional vs ambulatory blood pressure in older patients with systolic hypertension. Systolic Hypertension in Europe Trial Investigators. *JAMA : the journal of the American Medical Association* 1999; **282**(6): 539-46.

4. Ohkubo T, Hozawa A, Nagai K, et al. Prediction of stroke by ambulatory blood pressure monitoring versus screening blood pressure measurements in a general population: the Ohasama study. *Journal of hypertension* 2000; **18**(7): 847-54.

5. Mancia G, Zanchetti A, Agabiti-Rosei E, et al. Ambulatory blood pressure is superior to clinic blood pressure in predicting treatment-induced regression of left ventricular hypertrophy. SAMPLE Study Group. Study on Ambulatory Monitoring of Blood Pressure and Lisinopril Evaluation. *Circulation* 1997; **95**(6): 1464-70.

6. Imai Y, Ohkubo T, Sakuma M, et al. Predictive power of screening blood pressure, ambulatory blood pressure and blood pressure measured at home for overall and cardiovascular mortality: a prospective observation in a cohort from Ohasama, northern Japan. *Blood pressure monitoring* 1996; **1**(3): 251-4.

7. Fagard RH, Staessen JA, Thijs L. Prediction of cardiac structure and function by repeated clinic and ambulatory blood pressure. *Hypertension* 1997; **29**(1 Pt 1): 22-9.

8. Schettini C, Bianchi M, Nieto F, Sandoya E, Senra H. Ambulatory blood pressure: normality and comparison with other measurements. Hypertension Working Group. *Hypertension* 1999; **34**(4 Pt 2): 818-25.

9. Rasmussen SL, Torp-Pedersen C, Borch-Johnsen K, Ibsen H. Normal values for ambulatory blood pressure and differences between casual blood pressure and ambulatory blood pressure: results from a Danish population survey. *Journal of hypertension* 1998; **16**(10): 1415-24.

10. Mancia G, Sega R, Bravi C, et al. Ambulatory blood pressure normality: results from the PAMELA study. *Journal of hypertension* 1995; **13**(12 Pt 1): 1377-90.

11. Bjorklund K, Lind L, Lithell H. Twenty-four hour ambulatory blood pressure in a population of elderly men. *Journal of internal medicine* 2000; **248**(6): 501-10.

12. Pickering TG, Coats A, Mallion JM, Mancia G, Verdecchia P. Blood Pressure Monitoring. Task force V: White-coat hypertension. *Blood pressure monitoring* 1999; **4**(6): 333-41.

13. National Clinical Guideline C. National Institute for Health and Clinical Excellence Guidance CG127. Hypertension: The Clinical Management of Primary Hypertension in Adults: Update of Clinical Guidelines 18 and 34. London: Royal College of Physicians (UK); 2011.

14. Mancia G, Fagard R, Narkiewicz K, et al. 2013 ESH/ESC guidelines for the management of arterial hypertension: the Task Force for the Management of Arterial Hypertension of the European Society of Hypertension (ESH) and of the European Society of Cardiology (ESC). *European heart journal* 2013; **34**(28): 2159-219.

15. Pickering TG, Davidson K, Gerin W, Schwartz JE. Masked hypertension. *Hypertension* 2002; **40**(6): 795-6.

16. Sega R, Trocino G, Lanzarotti A, et al. Alterations of cardiac structure in patients with isolated office, ambulatory, or home hypertension: Data from the general population (Pressione Arteriose Monitorate E Loro Associazioni [PAMELA] Study). *Circulation* 2001; **104**(12): 1385-92.

17. Liu JE, Roman MJ, Pini R, Schwartz JE, Pickering TG, Devereux RB. Cardiac and arterial target organ damage in adults with elevated ambulatory and normal office blood pressure. *Annals of internal medicine* 1999; **131**(8): 564-72.

18. Ohkubo T, Kikuya M, Metoki H, et al. Prognosis of "masked" hypertension and "white-coat" hypertension detected by 24-h ambulatory blood pressure monitoring 10-year follow-up from the Ohasama study. *Journal of the American College of Cardiology* 2005; **46**(3): 508-15.

19. Mancia G, Facchetti R, Bombelli M, Grassi G, Sega R. Long-term risk of mortality associated with selective and combined elevation in office, home, and ambulatory blood pressure. *Hypertension* 2006; **47**(5): 846-53.

20. Tsai PS. White coat hypertension: understanding the concept and examining the significance. *Journal of clinical nursing* 2002; **11**(6): 715-22.

21. Longo D, Dorigatti F, Palatini P. Masked hypertension in adults. *Blood pressure monitoring* 2005; **10**(6): 307-10.

22. Whiting PF, Rutjes AW, Westwood ME, et al. QUADAS-2: a revised tool for the quality assessment of diagnostic accuracy studies. *Annals of internal medicine* 2011; **155**(8): 529-36.

23. Critical Appraisal Skills Programme (CASP). Cohort Study Checklist. <http://www.casp-uk.net/:> CASP UK; 2013.

24. Stroup DF, Berlin JA, Morton SC, et al. Meta-analysis of observational studies in epidemiology: a proposal for reporting. Meta-analysis Of Observational Studies in Epidemiology (MOOSE) group. *JAMA : the journal of the American Medical Association* 2000; **283**(15): 2008-12.

25. Abir-Khalil S, Zaimi S, Tazi MA, Bendahmane S, Bensaoud O, Benomar M. Prevalence and predictors of white-coat hypertension in a large database of ambulatory blood pressure monitoring. *Eastern Mediterranean Health Journal* 2009; **15**(2): 400-7.

26. Afsar B. The impact of different anthropometric measures on sustained normotension, white coat hypertension, masked hypertension, and sustained hypertension in patients with type 2 diabetes. *Endocrinology and Metabolism* 2013; **28**(3): 199-206.

27. Akilli H, Kayrak M, Aribas A, et al. The relationship between exercise capacity and masked hypertension in sedentary patients with diabetes mellitus. *Clinical & Experimental Hypertension (New York)* 2014; **36**(1): 9-16.

28. Andalib A, Akhtari S, Rigal R, et al. Determinants of masked hypertension in hypertensive patients treated in a primary care setting. *Internal Medicine Journal* 2012; **42**(3): 260-6.

29. Asayama K, Sato A, Ohkubo T, et al. The association between masked hypertension and waist circumference as an obesity-related anthropometric index for metabolic syndrome: the Ohasama study. *Hypertension Research - Clinical & Experimental* 2009; **32**(6): 438-43.

30. Azizi GBE, Ahid S, Abir-Khalil S, et al. Prevalence of masked hypertension in Morocco in the antecedents of hypertension. *Pharmacoepidemiology and Drug Safety* 2013; **22**: 45-6.

31. Bakalakou E, Kyfnidis K, Kallistratos E, et al. The effect of isolated masked nocturnal hypertension on arterial stiffness. *European heart journal* 2013; **34**: 1050.

32. Barochiner J, Cuffaro PE, Aparicio LS, et al. Predictors of masked hypertension among treated hypertensive patients: an interesting association with orthostatic hypertension. *American Journal of Hypertension* 2013; **26**(7): 872-8.

33. Ben-Dov IZ, Mekler J, Ben-Arie L, Bursztyn M. Lack of association between body-mass index and white-coat hypertension among referred patients. *Blood pressure monitoring* 2007; **12**(2): 95-9.

34. Ben-Dov IZ, Ben-Ishay D, Mekler J, Ben-Arie L, Bursztyn M. Increased prevalence of masked blood pressure elevations in treated diabetic subjects. *Archives of Internal Medicine* 2007; **167**(19): 2139-42.

35. Bucio JFC, Hernandez GS, Soriano RE. Obesity as a risk factor of white coat hypertension in ambulatory patients. [Spanish] La obesidad como factor de riesgo de la hipertension de bata blanca en pacientes de consulta externa. *Medicina Interna de Mexico* 2011; **27**(1): 11-6.

36. Cacciolati C, Hanon O, Alperovitch A, Dufouil C, Tzourio C. Masked hypertension in the elderly: cross-sectional analysis of a population-based sample. *American Journal of Hypertension* 2011; **24**(6): 674-80.

37. Calvo-Vargas CG, Gutierrez CG, Flores-Lopez AA, Parra-Rodriguez L, Polanco-Preza MA. "White Coat" effect associated factors detected with the loaned self-measurement equipment model. *American Journal of Hypertension* 1999; **12**(4): 153A-A.

38. Charvat J, Chlumsky J, Szabo M, Zamrazil V. The prevalence of masked hypertension in treated type 2 diabetic patients and its association with cardiovascular target organ damage. *Atherosclerosis Supplements* 2010; **11 (2)**: 158-9.

39. Dolan E, Stanton A, Atkins N, et al. Determinants of white-coat hypertension. *Blood pressure monitoring* 2004; **9**(6): 307-9.

40. Florian R, Stanley SF, David L, Ronald GV. Increased Prevalence of Masked Hypertension in Blacks. *Journal of Clinical Hypertension* 2013; **15**.

41. Gorostidi M, Sarafidis PA, de la Sierra A, et al. Differences between office and 24-hour blood pressure control in hypertensive patients with CKD: A 5,693-patient cross-sectional analysis from Spain. *American Journal of Kidney Diseases* 2013; **62**(2): 285-94.

42. Gualdiero P, Niebauer J, Addison C, Clark SJ, Coats AJ. Clinical features, anthropometric characteristics, and racial influences on the 'white-coat effect' in a single-centre cohort of 1553 consecutive subjects undergoing routine ambulatory blood pressure monitoring. *Blood pressure monitoring* 2000; **5**(2): 53-7.

43. Hanninen MR, Niiranen TJ, Puukka PJ, Mattila AK, Jula AM. Determinants of masked hypertension in the general population: the Finn-Home study. *Journal of hypertension* 2011; **29**(10): 1880-8.

44. Hermida RC, Ayala DE, Calvo C, et al. Factors infuencing the difference between clinic and ambulatory blood pressure measurements in untreated patients with essential hypertension. *American Journal of Hypertension* 2004; **17**(5): 42A-A.

45. Hernandez del Rey R, Armario P, Sanchez P, et al. [Frequency of white coat arterial hypertension in mild hypertension. Profile of cardiovascular risk and early organic involvement]. *Medicina Clinica* 1996; **106**(18): 690-4.

46. Hiraizumi T, Kumano H, Munakata M, Yoshinaga K, Taguchi F, Yamauchi Y. Autonomic nervous functions and psycho-behavioral factors associated with white coat phenomenon in hypertensive patients. [Japanese]. *Japanese Journal of Psychosomatic Medicine* 1998; **38**(6): 397-405.

47. Horikawa T, Obara T, Ohkubo T, et al. Difference between home and office blood pressures among treated hypertensive patients from the Japan Home versus Office Blood Pressure Measurement Evaluation (J-HOME) study. *Hypertension Research - Clinical & Experimental* 2008; **31**(6): 1115-23.

48. Hozawa A, Ohkubo T, Nagai K, et al. Factors affecting the difference between screening and home blood pressure measurements: The Ohasama Study. *Journal of hypertension* 2001; **19**(1): 13-9.

49. Huang CC, Wu TC, Lin SJ, Chen JW, Leu HB. Clinical predictors of significant white-coat effect in non-diabetic hypertensive patients. *Acta Cardiologica Sinica* 2010; **26**(3): 151-6.

50. Hwang ES, Choi KJ, Kang DH, et al. Prevalence, predictive factor, and clinical significance of white-coat hypertension and masked hypertension in Korean hypertensive patients. *Korean Journal of Internal Medicine* 2007; **22**(4): 256-62.

51. Iimuro S, Imai E, Watanabe T, et al. Clinical correlates of ambulatory BP monitoring among patients with CKD. *Clinical Journal of The American Society of Nephrology: CJASN* 2013; **8**(5): 721-30.

52. Ishikawa J, Kario K, Eguchi K, et al. Erratum: Regular alcohol drinking is a determinant of masked morning hypertension detected by home blood pressure monitoring in medicated hypertensive patients with well-controlled clinic blood pressure: The Jichi Morning Hypertension Research (J-MORE) study (Hypertension Research (2006) vol. 29 (679-686)). *Hypertension Research* 2007; **30**(2): 203.

53. Jhalani J, Goyal T, Clemow L, Schwartz JE, Pickering TG, Gerin W. Anxiety and outcome expectations predict the white-coat effect. *Blood pressure monitoring* 2005; **10**(6): 317-9.

54. Kabutoya T, Ishikawa J, Hoshide S, et al. Determinants of negative white-coat effect in treated hypertensive patients: the Jichi Morning Hypertension Research (J-MORE) study. *American Journal of Hypertension* 2009; **22**(1): 35-40.

55. Kayrak M, Bacaksiz A, Vatankulu MA, et al. Exaggerated blood pressure response to exercise--a new portent of masked hypertension. *Clinical & Experimental Hypertension (New York)* 2010; **32**(8): 560-8.

56. Kim S, Bae J. Pulse wave velocity and carotid intima media thickeness between masked hypertension, sustained hypertension and normotension in a worksite. *International Journal of Cardiology* 2011; **152**: S95.

57. Koupil I, Leon DA, Byberg L. Birth weight, hypertension and "white coat" hypertension: size at birth in relation to office and 24-h ambulatory blood pressure. *Journal of Human Hypertension* 2005; **19**(8): 635-42.

58. Labinson PT, Giacco S, Gift H, Mansoor GA, White WB. The importance of the clinical observer in the development of a white-coat effect in African-American patients with hypertension. *Blood pressure monitoring* 2008; **13**(3): 139-42.

59. Lee HY, Park JB. Prevalence and risk factors of masked hypertension identified by multiple self-blood pressure measurement. *Hypertension* 2008; **52**(5): e137-8; author reply e9.

60. Lerman CE, Brody DS, Hui T, Lazaro C, Smith DG, Blum MJ. The white-coat hypertension response: prevalence and predictors. *Journal of General Internal Medicine* 1989; **4**(3): 226-31.

61. Lindbaek M, Sandvik E, Liodden K, Mjell J, Ravnsborg-Gjertsen K. Predictors for the white coat effect in general practice patients with suspected and treated hypertension. *British Journal of General Practice* 2003; **53**(495): 790-3.

62. MacDonald MB, Laing GP, Wilson MP, Wilson TW. Prevalence and predictors of white-coat response in patients with treated hypertension. *CMAJ Canadian Medical Association Journal* 1999; **161**(3): 265-9.

63. Mallion JM, Clerson P, Bobrie G, Genes N, Vaisse B, Chatellier G. Predictive factors for masked hypertension within a population of controlled hypertensives. *Journal of hypertension* 2006; **24**(12): 2365-70.

64. Manios ED, Koroboki EA, Tsivgoulis GK, et al. Factors influencing white-coat effect. *American Journal of Hypertension* 2008; **21**(2): 153-8.

65. Mansoor GA, McCabe EJ, White WB. Determinants of the white-coat effect in hypertensive subjects. *Journal of Human Hypertension* 1996; **10**(2): 87-92.

66. Makris TK, Thomopoulos C, Papadopoulos DP, et al. Association of passive smoking with masked hypertension in clinically normotensive nonsmokers. *American Journal of Hypertension* 2009; **22**(8): 853-9.

67. Martinez MA, Garcia-Puig J, Martin JC, et al. Frequency and determinants of white coat hypertension in mild to moderate hypertension: a primary care-based study. Monitorizacion Ambulatoria de la Presion Arterial (MAPA)-Area 5 Working Group. *American Journal of Hypertension* 1999; **12**(3): 251-9.

68. Nasothimiou EG, Tzamouranis D, Rarra V, Roussias LG, Stergiou GS. Diagnostic accuracy of home vs. ambulatory blood pressure monitoring in untreated and treated hypertension. *Hypertension research : official journal of the Japanese Society of Hypertension* 2012; **35**(7): 750-5.

69. Niiranen TJ, Jula AM, Kantola IM, Reunanen A. Prevalence and determinants of isolated clinic hypertension in the Finnish population: the Finn-HOME study. *Journal of hypertension* 2006; **24**(3): 463-70.

70. Obara T, Ohkubo T, Funahashi J, et al. Isolated uncontrolled hypertension at home and in the office among treated hypertensive patients from the J-HOME study. *Journal of hypertension* 2005; **23**(9): 1653-60.

71. Parati G, Omboni S, Stergiou G, et al. Geographical features and determinants of masked hypertension in 9753 hypertensive subjects from five continents: The artemis international registry. *High Blood Pressure and Cardiovascular Prevention* 2012; **19 (3)**: 145.

72. Park SJ, Park JB, Choi DJ, et al. Detection of masked hypertension and the 'mask effect' in patients with well-controlled office blood pressure. *Circulation Journal* 2011; **75**(2): 357-65.

73. Rasmussen SL, Torp-Pedersen C, Borch-Johnsen K, Ibsen H. Normal values for ambulatory blood pressure and differences between casual blood pressure and ambulatory blood pressure: Results from a Danish population survey. *Journal of hypertension* 1998; **16**(10): 1415-24.

74. Rodrigues TC, Leitao CB, Kramer CK, et al. The "white-coat effect" is more relevant at higher office blood pressure levels. *Diabetes* 2009; **58**.

75. Sandvik E, Steine S. White coat hypertension in a general practice. Prevalence, cardiovascular risk factors and clinical implications. *Scandinavian Journal of Primary Health Care* 1998; **16**(4): 222-6.

76. Schoenthaler AM, Schwartz J, Cassells A, Tobin JN, Brondolo E. Daily interpersonal conflict predicts masked hypertension in an urban sample. *American Journal of Hypertension* 2010; **23**(10): 1082-8.

77. Sheppard JP, Holder R, Nichols L, et al. Predicting out-of-office blood pressure level using repeated measurements in the clinic: an observational cohort study. *Journal of hypertension* 2014; **32**(11): 2171-8; discussion 8.

78. Smirnova MI, Gorbunov VM, Deev AD, Andreeva GF. PREDICTORS OF MASKED HYPERTENSION IN TREATED PATIENTS. *Journal of hypertension* 2009; **27**: S48-S.

79. Sobrino J, Domenech M, Camafort M, Vinyoles E, Coca A, investigators Eg. Prevalence of masked hypertension and associated factors in normotensive healthcare workers. *Blood pressure monitoring* 2013; **18**(6): 326-31.

80. Sobrino J, Domenech M, Camafort M, Vinyoles E, Coca A, investigadores del grupo E. [Prevalence of masked hypertension in a cohort of controlled hypertensive patients in Spain]. *Medicina Clinica* 2011; **136**(14): 607-12.

81. Spruill TM, Pickering TG, Schwartz JE, et al. The impact of perceived hypertension status on anxiety and the white coat effect. *Annals of Behavioral Medicine* 2007; **34**(1): 1-9.

82. Streitel KL, Graham JE, Pickering TG, Gerin W. Explaining gender differences in the white coat effect. *Blood pressure monitoring* 2011; **16**(1): 1-6.

83. Sung SH, Cheng HM, Wang KL, et al. White coat hypertension is more risky than prehypertension: important role of arterial wave reflections. *Hypertension* 2013; **61**(6): 1346-53.

84. Tam TKW, Ng KK, Lau CM. What are the predictors of white-coat hypertension in Chinese adults? *Hong Kong Practitioner* 2007; **29**(11): 411-8.

85. Tardif JC, Curnew GP, Rigal R, Vaillancourt M. Determinants of Masked Hypertension in Hypertensive Patients Treated in the Real-Life Observational DioVantageIV Study. *Circulation* 2009; **120**(18): S474-S5.

86. Thomas O, Day K, Martin U, Thomas M, Dasgupta I. White coat hypertension in treated hypertensive patients and factors influencing it. *Journal of Human Hypertension* 2012; **26**(10): 618-.

87. Trudel X, Brisson C, Larocque B, Milot A. Masked hypertension: different blood pressure measurement methodology and risk factors in a working population. *Journal of hypertension* 2009; **27**(8): 1560-7.

88. Tsai PS. Determinants of the white-coat effect in normotensives and never-treated mild hypertensives. *Clinical & Experimental Hypertension (New York)* 2003; **25**(7): 443-54.

89. Uzu T, Nakao K, Kume S, et al. High sodium intake is associated with masked hypertension in Japanese patients with type 2 diabetes and treated hypertension. *American Journal of Hypertension* 2012; **25**(11): 1170-4.

90. Verdecchia P, Palatini P, Schillaci G, Mormino P, Porcellati C, Pessina AC. Independent predictors of isolated clinic ('white-coat') hypertension. *Journal of hypertension* 2001; **19**(6): 1015-20.

91. Wang GL, Li Y, Staessen JA, Lu L, Wang JG. Anthropometric and lifestyle factors associated with white-coat, masked and sustained hypertension in a Chinese population. *Journal of hypertension* 2007; **25**(12): 2398-405.

92. Wing LM, Brown MA, Beilin LJ, et al. 'Reverse white-coat hypertension' in older hypertensives. *Journal of hypertension* 2002; **20**(4): 639-44.

93. Yoon HJ, Ahn Y, Kim KH, et al. Can pulse pressure predict the white-coat effect in treated hypertensive patients? *Clinical & Experimental Hypertension (New York)* 2012; **34**(8): 555-60.

94. Zhou J, Liu C, Shan P, Zhou Y, Xu E, Ji Y. Prevalence and distinguishing features of masked hypertension in type 2 diabetic patients. *Journal of Diabetes & its Complications* 2013; **27**(1): 82-6.
